# Protecting boreal caribou habitat can help conserve biodiversity and safeguard large quantities of soil carbon in Canada

**DOI:** 10.1038/s41598-022-21476-x

**Published:** 2022-10-12

**Authors:** Cheryl A. Johnson, C. Ronnie Drever, Patrick Kirby, Erin Neave, Amanda E. Martin

**Affiliations:** 1 Environment and Climate Change Canada, Science and Technology, National Wildlife Research Centre, Ottawa, ON K1A 0H3 Canada; 2 Department of Applied Geomatics, University of Sherbrooke, Sherbrooke, QC J1K 2R1 Canada; 3 Nature United, Ottawa, ON Canada; 4 Department of Biology, Carleton University, Ottawa, ON K1S 5B6 Canada

**Keywords:** Ecology, Biodiversity, Boreal ecology, Conservation biology

## Abstract

Boreal caribou require large areas of undisturbed habitat for persistence. They are listed as threatened with the risk of extinction in Canada because of landscape changes induced by human activities and resource extraction. Here we ask: Can the protection of habitat for boreal caribou help Canada meet its commitments under the United Nations Convention on Biological Diversity and United Nations Framework Convention on Climate Change? We identified hotspots of high conservation value within the distribution of boreal caribou based on: (1) three measures of biodiversity for at risk species (species richness, unique species and taxonomic diversity); (2) climate refugia or areas forecasted to remain unchanged under climate change; and, (3) areas of high soil carbon that could add to Canada’s greenhouse gas emissions if released into the atmosphere. We evaluated the overlap among hotspot types and how well hotspots were represented in Canada’s protected and conserved areas network. While hotspots are widely distributed across the boreal caribou distribution, with nearly 80% of the area falling within at least one hotspot type, only 3% of the distribution overlaps three or more hotspots. Moreover, the protected and conserved areas network only captures about 10% of all hotspots within the boreal caribou distribution. While the protected and conserved areas network adequately represents hotspots with high numbers of at risk species, areas occupied by unique species, as well as the full spectrum of areas occupied by different taxa, are underrepresented. Climate refugia and soil carbon hotspots also occur at lower percentages than expected. These findings illustrate the potential co-benefits of habitat protection for caribou to biodiversity and ecosystem services and suggest caribou may be a good proxy for future protected areas planning and for developing effective conservation strategies in regional assessments.

## Introduction

The word crisis has become synonymous with climate change in mainstream media. Yet, it also aptly describes the loss of global biodiversity. Experts estimate that current rates and magnitudes of species losses are similar to or exceed those from the last five mass extinction events^1–3^. The Intergovernmental Science-Policy Platform on Biodiversity and Ecosystem Services, the biodiversity equivalent of the International Panel on Climate Change, estimates that over half a million terrestrial species currently lack sufficient habitat for persistence^3^. Millions more species may go extinct in the coming decades without the transformative economic, social and political changes required to address the underlying issues. Land use change, resource development and climate change, which are often interdependent and interact in complex ways, are among the top direct drivers of the loss of biodiversity and ecosystem services across the globe^4^. Experts have raised serious concerns about the current capacity and resilience of ecosystems to adjust to the environmental changes projected with climate change^5,6^.

The twin crises of biodiversity loss and climate change have precipitated international calls for the expansion of protected areas networks. In 2010, the Parties to the United Nations Convention on Biological Diversity (UNCBD) endorsed a strategic plan to halt the global decline of biodiversity that involved 20 targets, one of which was protecting at least 17% of terrestrial and inland waters by 2020 (Aichi target 11)^7^. A new target of 30% protection by 2030 is under consideration. Similarly, the United Nations Framework Convention on Climate Change (UNFCCC) recognizes habitat protection as an important mechanism for reducing the estimated 12–25% of global greenhouse gas emissions caused by the release of carbon into the atmosphere from land conversion and land degradation^8^.

There is increased recognition for the need to accommodate for climate change in protected areas planning and management on top of the typical considerations, such as human land use, rare and endangered species, and exotics^6,9,10^. Several studies in tropical forests of Africa, Asia, Central and South America, and Madagascar have examined the co-benefits of carbon stock conservation to biodiversity for protected areas planning^11–13^. Climate refugia, defined as areas more likely to experience similar future climate conditions, limit biodiversity loss by providing opportunities for species to respond to spatial shifts in climatic conditions under climate warming^14,15^. Climate refugia are increasingly being used in conjunction with more common metrics, such as, species richness, endemic or unique species, or taxonomic diversity, to identify important areas for biological conservation^15,16^. For example, Carrol & Ray suggest that comparisons of the commonalities and contrasts between biodiversity, carbon-rich areas and climate refugia could enhance protected and conserved areas networks across North America to address biodiversity and climate change simultaneously^16^.

As one of the largest countries by area among the Parties to the UNCBD and UNFCCC^10^_,_ with large carbon stocks found in expansive, intact peatlands and old-growth forests^17,18^, Canada has the potential to influence how other governments around the globe augment and enhance their protected and conserved areas networks. Canada fell short of meeting the 2020 Aichi target by protecting < 13%^19^. Similarly, it has failed to achieve several of its commitments to reduce greenhouse gas emissions over the last two decades^20^. Canada could benefit from examples of strategic and effective placement of new protected and conserved areas that consider the interrelated pathways between its policies on biodiversity and climate change simultaneously^5,6,9,10^ in preparation for the upcoming post-2020 UNCBD discussions in this winter.

**Figure 1:**
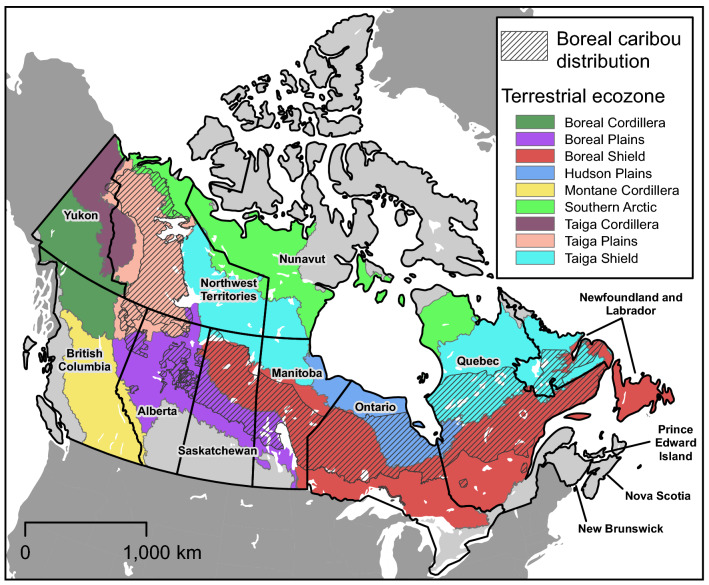
The distribution of boreal caribou in Canada. Boundaries were updated from the 2011 boundaries^20^ to include additional areas identified by the provinces and territories in 2015.

Landscape initiatives that fulfill the legal obligation to protect critical habitat for boreal woodland caribou, *Rangifer tarandus caribou* (hereafter boreal caribou), have the potential to protect biodiversity and ecosystem services at the continental scale in Canada^21,22^. These caribou range over > 2.4 million km^2^ of the boreal forest of Canada from the Yukon to the Atlantic coast (Fig. 1). Boreal caribou require large tracts of undisturbed old growth forest or wetland complexes to separate themselves spatially from predators, like wolves (*Canis lupus*) and black bears (*Ursus americanus*)^23^. Land use activities in the boreal forest of Canada have accelerated over the last few decades and are expected to increase into the near future^24,25^. These changes have not only exacerbated the impacts of climate change, but also altered the dynamics between boreal caribou and their predators and resulted in population declines that have led to the listing of the species as threatened with extinction under Canada’s Species at Risk Act (SARA)^26,27^. In 2012, about 65% of the habitat across the majority of boreal caribou local populations (~ 1.5 million km^2^ of the boreal forest) was designated as critical habitat. SARA imposes a legal obligation to effectively protect critical habitat, which, for boreal caribou, means managing and maintaining a state of 65% undisturbed habitat in each of the 51 local populations across the species’ distribution^28^. Unfortunately, habitat degradation, mainly from logging, mining and oil and natural gas activities, has continued since the designation of boreal caribou critical habitat^29^.

While several authors have focused on the socio-economic cost to protecting boreal caribou habitat^31,32^, few have investigated the potential co-benefits to biodiversity or efforts to address climate change^10,25^. Integrating the protection of large tracts of habitat for boreal caribou into the expansion of Canada’s protected and conserved areas network to 30% by 2030^19^ could help ensure the functioning of ecological processes across the Canadian boreal forest, including those related to evolutionary adaptation^10,24^. While the boreal forest is neither the most diverse biome nor the biome most affected by climate change in Canada, it has significant conservation value^10,25^. Protection of large tracts of undisturbed habitat for boreal caribou could benefit large populations of songbirds and other species including those at risk^24,25,32^. It could safeguard multiple species persistence under a variety of climate scenarios by protecting climate refugia^5,16^. Protection of peatland and old forest could also contribute to Canada’s strategy to reduce greenhouse gas emissions by safeguarding existing carbon stores^18,33^. The boreal biome currently stores one third of the world’s soil carbon^10,24^, where soil carbon accounts for nearly five times the carbon content of above ground biomass, or 85% of all total carbon in the boreal ecosystem^34^. In short, strategic planning for the expansion of Canada’s protected areas network focused on the protection of critical habitat for boreal caribou could help Canada meet: its domestic commitments for other at risk species^22^; its international commitments to reverse the loss of biodiversity under the UNCBD^21^; and, its international commitments under the UNFCCC to reduce greenhouse gas emissions by safeguarding existing carbon stores^33^.

We use hotspots and gap analyses^35^ to determine the extent to which the legal requirement to protect critical habitat for boreal caribou can help with strategic protected and conserved areas planning in Canada. We identify hotspots of high conservation value across the distribution of boreal caribou: (1) for three different measures of biodiversity for at risk species; (2) for climate refugia; and, (3) for soil carbon. We focus on at risk species threatened by land conversion and land degradation because these species are most likely to benefit from protection of critical habitat for boreal caribou and will have an immediate impact on reducing biodiversity loss in Canada. We compare the representation of hotspots in the 2019 protected and conserved areas network^36^ to that which is representative across the boreal caribou distribution in Canada. We measure spatial congruency among the different hotspots as an initial prioritization of potential areas for future protected areas planning. The results are evaluated in terms of the value of boreal caribou as a surrogate for achieving Canada’s biodiversity commitments and commitments to reduce greenhouse gas emissions by safeguarding globally important quantities of soil carbon.

## Results

### Hotspots

**Figure 2:**
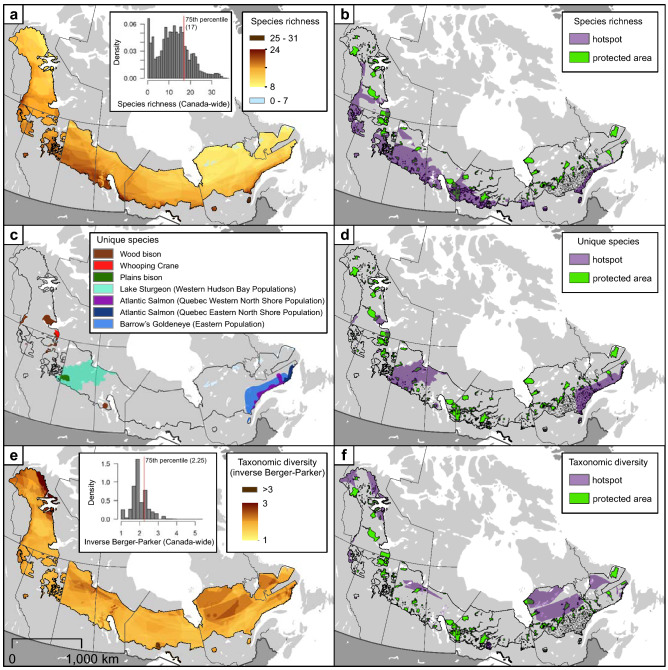
Distribution of raw values (left column) and hotspots (right column) for (**a**–**b**) species richness; (**c**–**d**) unique species; and (**e**–**f**) taxonomic diversity across the distribution of boreal caribou. Species richness and taxonomic diversity hotspots identified using top quantiles. Unique species hotspots identified as any area occupied by one of the seven species classified as unique within the boreal caribou distribution.

Hotspots were distributed widely, covering a total of ~ 80% of the 2, 440, 837 km^2^ of the boreal caribou distribution (Table 1; Fig. 2). Coverage for individual hotspots ranged from as low as ~ 14% for unique species up to a maximum of ~ 36% for soil carbon storage.

**Table 1:** Total area (km^2^) and area within the protected and conserved areas network for each hotspot across the boreal caribou distribution in Canada.

Hotspot	Total area of hotspot across the boreal caribou distribution in km^2^	Total area of hotspot in the protected and conserved areas network in km^2^
Species richness	613, 424 (25.13)	73, 491 (28.86)
Taxonomic diversity	425, 462 (17.43)	33, 196 (13.04)
Unique species	351, 587 (14.40)	36, 305 (14.26)
Climate refugia	476, 720 (19.53)	47, 583 (18.69)
Soil carbon storage	875, 376 (35.86)	86, 572 (34.00)

Values in brackets represent percentages across the boreal caribou distribution and protected and conserved areas network, respectively.

Species richness hotspots included between 17 and 31 at risk species. They covered ~ 25% of our study area (Table 1). Species richness showed the typical latitudinal and longitudinal gradient seen in the northern hemisphere with a high concentration of hotspots at the southern periphery of the boreal caribou distribution and in the west, more specifically in British Columbia, Alberta and Northwest Territories (Fig. 2a,b). In contrast, hotspots of high taxonomic diversity were located primarily at northern latitudes, while unique species hotspots were concentrated in the boreal plain and boreal shield of Saskatchewan (Lake Sturgeon, *Acipenser fulvescens*) and Manitoba (Wood Bison, *Bison bison athabascae*) as well as the eastern boreal shield of Québec (Atlantic Salmon, *Salmo salar* and Barrow’s Goldeneye, *Bucephala islandica*; Fig. 2c–f).

**Figure 3:**
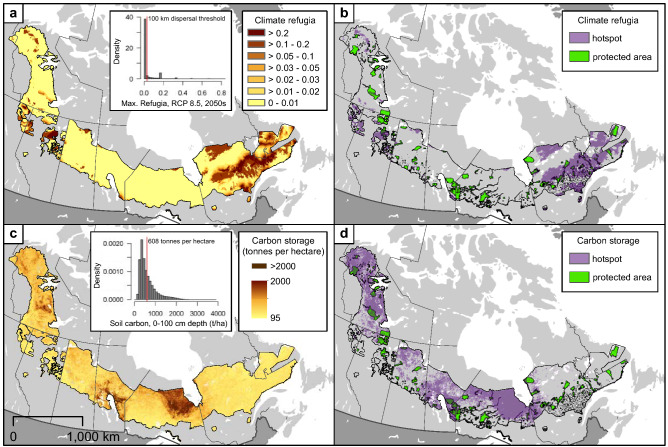
Spatial distribution of raw values (left column) and hotspots (right column) for (**a**–**b**) climate refugia; and (**c**–**d**) soil carbon storage across the distribution of boreal caribou. Climate refugia hotspots identified using a dispersal threshold of 100 km/century. Soil carbon storage hotspots identified as ≥ 608 tonnes/ha.

Coverage by climate refugia was relatively low across much of the distribution of boreal caribou (19.5%; Table 1), indicating divergence between present and future climates^15^ (Fig. 3a,b). The majority of areas that met the 100 km/century dispersal threshold used to identify hotspots were concentrated in Québec/Labrador in the east and British Columbia, Alberta, and the Northwest Territories in the west. By contrast, areas with ≥ 608 tonnes of soil carbon per hectare—approximately the 75% percentile of soil carbon density in the North American Boreal and among the highest densities of soil carbon on Earth^37^—covered a broad range of latitudes and longitudes within the distribution of boreal caribou (Fig. 3c,d).

### Gap analysis of hotspots in the protected and conserved areas network in Canada

**Figure 4:**
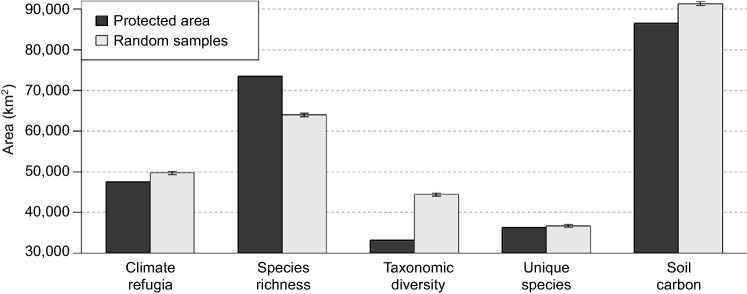
Gap analysis to quantify whether the area of the hotspots within the protected and conserved areas network (dark grey) is consistent with the area expected based on availability across the boreal caribou distribution (light grey) ± 95% confidence intervals. Availability determined from random sampling without replacement.

The protected and conserved areas network covered ~ 10% (254, 646 km^2^) of the boreal caribou distribution. While all hotspots were represented (Table 1), the gap analysis suggested most were under-represented in the protected and conserved areas network when compared to their availabilities across the boreal caribou distribution. Specifically, the network contained smaller areas of hotspots for climate refugia, taxonomic diversity, unique species and soil carbon storage than expected (Fig. 4). Hotspots for richness of at risk species were the exception. These were over-represented in the protected and conserved areas network compared to their occurrence across boreal caribou distribution (Fig. 4).

**Figure 5:**
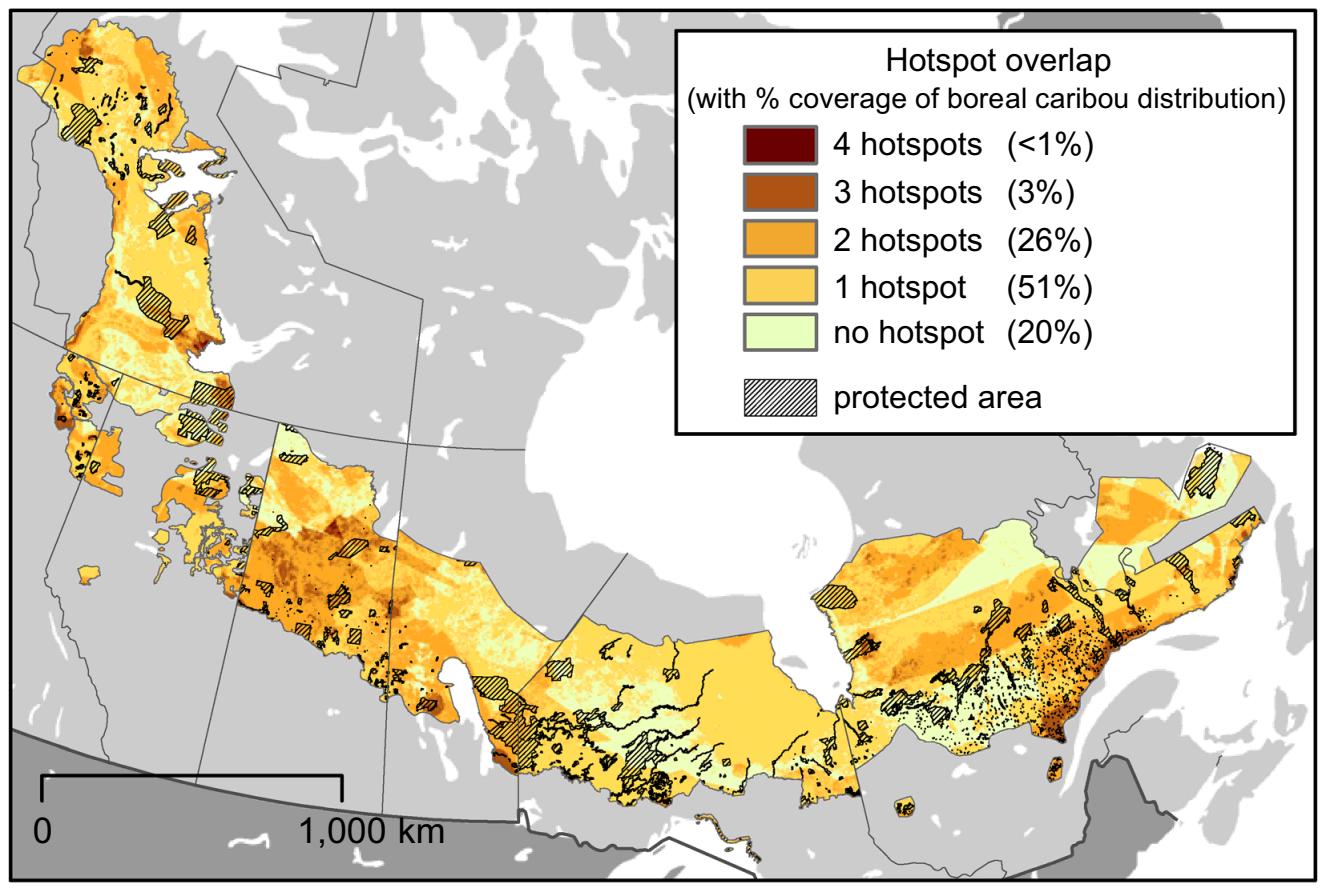
Spatial overlap among the different hotspots across the distribution of boreal caribou. The hatched areas show the existing protected and conserved areas network.

Most hotspots within the boreal caribou distribution occurred outside the protected and conserved areas network, with limited overlap (Fig. 5). Overlapping hotspots occupied less than 30% of our study area (≥ 2 hotspots/cell). Most overlaps were of only two hotspots (26%). Very few overlaps of four or five hotpots occurred. Most hotspot pairs overlapped less than that expected based on their occurrence (Table 2), with some exceptions. The positive Centered Jaccard Similarity Coefficient suggested species richness-unique species hotspots and taxonomic diversity-climate refugia hotspots were the most likely to co-occur. Figures 2b,d,f and 3b highlight potential areas of interest in Québec and Labrador. Areas of high species richness co-occur with the Atlantic salmon and Barrow’s Goldeneye (unique species) along the eastern coast of Québec and Labrador, whereas climate refugia and areas with proportional more taxa co-occur further to the north and extend further to the west.

**Table 2:** Pairwise comparison of overlap between hotspots across the boreal caribou distribution.

Pairwise comparison of spatial overlap between hotspots		Centered Jaccard Similarity Coefficient
Species Richness	Taxonomic Diversity	− 0.100
***Species Richness***	***Unique Species***	***0.125***
Species Richness	Climate Refugia	− 0.035
Species Richness	Soil Carbon Storage	− 0.061
Taxonomic Diversity	Unique Species	− 0.037
***Taxonomic Diversity***	***Climate Refugia***	***0.103***
Taxonomic Diversity	Soil Carbon Storage	− 0.054
***Unique Species***	***Climate Refugia***	***0.007***
Unique Species	Soil Carbon Storage	− 0.070
Climate Refugia	Soil Carbon Storage	− 0.114

Comparisons in bold italics identify areas that overlap more than expected by random (positive Centered Jaccard Similarity Coefficients).

## Discussion

The success of planning for future protected areas depends on adequately capturing co-benefits to biodiversity and ecosystem services, including those that help address climate change^5,6,16^. We illustrate how the legal requirement to protect critical habitat for an at risk species, like boreal caribou, can serve as a proxy for multi-species and ecosystem conservation planning at a national scale^21,22^. Eighty percent or 1.8 million km^2^ of the boreal caribou distribution represent hotspots that could contribute to the conservation of biodiversity, climate refugia or carbon stocks while protecting critical habitat for the species. Moreover, our analyses revealed opportunities to extend these co-benefits to protected and conserved areas by showing that only about 10% of the hotspots across the boreal caribou distribution were protected formally and most were proportionally underrepresented in Canada’s current network. Like the co-benefits to biodiversity and carbon stock conservation achieved through Jaguar habitat protection in Brazil^11^, our study illustrates the potential for a wide-ranging, at risk species to help guide Canada’s protected area expansion efforts. Clearly, the distribution of boreal caribou does not include southern areas where biodiversity in Canada is most imperiled^10^ or northern areas of the Arctic where the effects of climate change are most pronounced. Nevertheless, boreal caribou do cover about one third of Canada’s landmass and, as such, can make a valuable contribution to strategic plans to bolster Canada’s protect areas network.

The protection of carbon stocks has been highlighted as an effective measure to reduce greenhouse gas emissions from tropical deforestation in Africa, Asia and South America^11,12^. In the boreal biome, predicted increases in the future frequency of large wildfires and pest infestations with climate warming will exacerbate future losses of carbon storage from land conversion^18,38^. Soil carbon hotspots covered ~ 36% of the distribution of boreal caribou, indicating the potential benefit of critical habitat protection in safeguarding carbon stocks. Strategic protection of boreal caribou critical habitat in the Northwest Territories, northern Saskatchewan and Manitoba could protect areas of high carbon as well as species richness hotspots or hotspots for both terrestrial and aquatic species unique to the distribution of boreal caribou, like Wood Bison, Plain Bison or Lake Sturgeon. Likewise, strategic protection of peatlands in the Hudson Bay Lowlands of northern Ontario for boreal caribou could protect below ground carbon stocks while simultaneously protecting important watersheds/hotspots for native fish species^39^. Little has been done to address climate and change in Canada’s protected areas planning and management over the last decade^6^. The proactive protection of carbon stock such as peatlands within the distribution of boreal caribou could help Canada address this gap^18^.

Climate refugia are essential to species persistence and the long-term maintenance of ecosystem function^12–14^ as species’ distributions shift, and community structure and composition change across the boreal forest under climate warming^38^. Strategic protection of large areas for boreal caribou in eastern Canada like south-central Québec and Labrador could bolster the representation of climate refugia in Canada’s protected and conserved areas network while enhancing the protection of hotspots for unique species (Atlantic Salmon and Barrow’s Goldeneye), at risk species, or hotspots with higher taxonomic diversity. Parts of south-central Québec extending to the border of Labrador have also been identified as national-scale hotspots for provision of ecological services related to freshwater^40^. Restricting our analyses to the distribution of caribou resulted in the omission of important climate refugia in southern Ontario; however, our analysis captured previously identified gaps in climate refugia across Saskatchewan, Manitoba and northern Ontario that indicate large expanses of the boreal forest in Canada may experience rapid northern shifts in climatic conditions^15^. Species in these areas are unlikely to be able to disperse to and colonize new areas with favourable climate conditions. Finding management solutions that help reduce the risk of species extinction may be difficult given the complex interactions across trophic levels in food webs. For example, land-use activities like salvage logging that help accelerate forest regrowth may further aggravate changes in caribou-moose-wolf interactions following disturbances from fire or pest infestation and lead to higher caribou predation^38,41^.

Similar to other studies^32,42^, our analysis characterized areas occupied by boreal caribou in northeastern British Columbia, and northeastern and northwestern Alberta as hotspots for at risk biodiversity. The high occurrence of at risk species is not surprising given these areas have among the highest habitat disturbance levels from oil and gas and other resource extraction activities within the boreal caribou distribution^29,30^. Although hotspots with high richness of at-risk species across the distribution of boreal caribou are well represented in Canada’s existing protected areas network, additional protection is needed to prevent species’ extirpations or extinctions and reduce biodiversity loss^10^. Enhanced protection in these regions would have the additional co-benefits of increasing representation of climate refugia, enhancing north–south and east–west connectivity in the protected areas network, and increasing representation of ecosystem diversity by incorporating western and southern ecoregions under-represented in the protected areas network^10^. In short, expansion of protected areas within the distribution of boreal caribou along the border of British Columbia and Alberta would satisfy four of the five key principles to biodiversity conservation^10^ with the exception of conserving intact wilderness areas because of the high levels of human disturbance in these regions^29^.

Our results also suggest that strategic planning will be required to optimize the co-benefits of habitat protection for caribou under any expansion of the existing protected areas network to achieve the 30% target by 2030. The lack of overlap among hotspots of biodiversity and ecosystem services is not a challenge unique to our study area or to the hotspots we examined^40,43^, re-emphasizing that not all efforts to expand protected areas would be equally effective^5^. Our analysis was not intended to be exhaustive, but rather to highlight how caribou can serve as a proxy for the protection of biodiversity and ecosystem services in boreal Canada. The resulting hotspot maps provide a simple decision support tool that, when used in conjunction with other tools developed using more regional information or examining different biodiversity measures and ecosystem services, can help decision makers evaluate the cost/benefits of protection of caribou habitat against other societal goals or constraints^5,10,16^.

In addition to a need for strategic planning, our work illustrates another common challenge in conservation decision-making: conflict with resource extraction or land use^4,10,40^. The discussion on caribou in Alberta and British Columbia has focused on the socio-economic costs of protecting and restoring areas heavily affected by human land use activities^30,31^. These costs have undoubtedly contributed to the lack of effective protection for boreal caribou critical habitat^29^. Similarly, rapid increases in human disturbances across boreal caribou ranges in Québec have caused experts to raise concern about the influence of foreign investments in resource extraction on decision making around habitat protection in Canada^31^. Considerably less focus has been placed on the value of these areas for biodiversity and ecosystem services or their value to Indigenous peoples, in terms of respecting their treaty rights and traditional ways of living. The boreal biome provides $703 billion annually in terms of ecosystem services alone^44^. The explicit representation of “natural capital”^25^ for biodiversity and ecosystem services and the cost to Indigenous peoples are needed to counterbalance the current focus on resource exploitation to more accurately reflect the trade-offs in socio-economic analyses^45^.

There are many ways to recognize and support Indigenous treaty rights in protected areas planning. For example, Indigenous Protected Areas, defined as Indigenous owned lands managed in accordance with Indigenous traditional laws, customs and culture that contribute to the long-term conservation of nature, make up > 40% of the protected areas network in Australia^46,47^. Alternatively, culturally significant areas prioritized by Indigenous people could be included as a spatial layer in formal strategic assessments informing decisions about new protected areas^48^. Community workshops are a good venue for discussing conservation priorities using participatory mapping approaches, where the priority areas are delineated on maps as a way of spatially representing Indigenous Knowledge. Community areas of interest might describe provisional services, such as areas valued for fishing or hunting, trap lines, or areas for harvesting medical plants, to name a few. On the other hand, Indigenous communities may prefer to focus on cultural services, referring to non-material benefits that contribute knowledge building, creativity, and the development and advancement of people. Focus on cultural services could allow for a better representation of the values, customs and principles of living that are an essential element of many Indigenous Knowledge systems. Cultural services are not widely used in conservation planning and decision making^40,48^.

Individual national conservation goals will ultimately determine how countries select areas to meet the Aichi target to protect 30% of land and waters by 2030. Our work on caribou highlights the potential for similar wide-ranging species to act as surrogates to achieve multiple, simultaneous conservation goals through the prioritization of areas for biodiversity and ecosystem services^11^. We identify some key challenges with achieving multiple conservation objectives. Overlap among conservation hotspots is often limited and areas of conservation co-benefits may be important for resource extraction or land use activities^10^. An optimization analysis^10,14^ that not only identifies areas that maximize co-benefits but also evaluates the trade-offs to achieving multiple goals would be a logical next step^49^. This national-scale analyses provides a starting point for regional assessments of the relative importance of biodiversity, ecosystem services, and biocultural elements, including Indigenous rights, tourism and/or recreation, to different partners and stakeholders; an essential next step to developing effective conservation strategies for boreal Canada and perhaps elsewhere.

## Methods

### Co-occurrence between boreal caribou and other at risk species

Our study area is the distribution of boreal caribou in Canada. We adjusted the 2011 boreal caribou distribution^28^ to include additional areas of occurrence from updated population range boundaries provided to Environment and Climate Change Canada by territorial and provincial jurisdictions in 2015 (Fig. 1).

We used the 2018 Species Assessment report published by the Committee on the Status of Endangered Wildlife in Canada^50^ (COSEWIC) to identify terrestrial and aquatic species or subspecies (N = 95; Supplementary Table S1) listed as Special Concern, Threatened or Endangered occurring within the distribution of boreal caribou. We used COSEWIC status assessments because they are based solely on knowledge of the species’ and, unlike official listing under the federal SARA, not influenced by socioeconomic or political considerations. We included in our analysis only species for which human disturbance was a threat to persistence to ensure the benefits of protecting 65% undisturbed habitat boreal caribou critical habitat (areas > 500 m from human without fire disturbance for ≥ 40 years^28^) applied. Dune grasses threatened by forest encroachment and migratory bird species threatened by food or habitat supply outside the Canadian boreal forest were removed. Arctic Grayling (*Thymullas arcticus*) was added to our species’ list because the fish is designated as Special Concern in Alberta, Canada, is experiencing population decline in other Canadian jurisdictions and is considered a high priority for future assessment (not currently listed) by COSEWIC^51^. In total, 80 species/subspecies from nine taxa met our criteria for inclusion (Table 3).

**Table 3:** Taxonomic representation of number of species/subspecies assessed as Endangered, Threatened or Special Concern by the COSEWIC across the boreal caribou distribution.

Taxonomic Group	Number of species or subspecies	COSEWIC species’ assessment		
		Endangered	Threatened	Special Concern
Amphibians	4	0	0	4
Arthropods	7	4	0	3
Birds	27	3	13	11
Freshwater and Anadromous Fish	18	3	10	5
Lichens	2	0	1	1
Mammals	14	5	3	6
Molluscs	1	0	1	0
Reptiles	3	0	0	3
Plants	4	2	0	2
Total	80	17	28	35

Species were included only if human disturbance was identified as a threat to persistence.

Existing geospatial data sets on extent of species occurrences^52^ were augmented by digitizing maps from species-specific assessments prepared by COSEWIC or the federal Department of Fisheries and Oceans (Supplementary Table S1 and Data S1). We clipped the extent of occurrence maps to the updated boreal caribou distribution. These data sets, along with all others used in this project, were represented (rasterized when needed) on a common analysis grid using an Albers equal-area projection of 1 km by 1 km cells to match the climate refugia data that represented the coarsest resolution in our analyses (see below). We assigned a value of “1” to any 1 km cell if a species occupied ≥ 50% of the cell and “0” to designate unoccupied cell (< 50%).

We calculated biodiversity hotspots using three measures of biodiversity^53^. The measures were selected based on the ease with which they could be interpreted. First, we calculated Species Richness as the sum of all species within each 1 km cell (Supplemental Data S2). Next, we calculated taxonomic diversity (TD) to assess evenness or heterogeneity due to lack of information on species’ abundances. We used a modified version of the inverse of the Berger-Parker index^53^ to measure taxonomic heterogeneity (Eq. 1):

1 $$TD = \frac{\# of\;species\;in\;cell\;i}{{\# of\;species\;from\;the\;most\;dominant\;taxon\;in\;cell\;i}}$$

where *i* indexes 1 km^2^ cells within the boreal caribou distribution (Supplemental Data S3). Taxonomic heterogeneity increases with *TD*. Our third biodiversity measure was unique species. Seven species were identified as relatively unique or occupying restricted geographical areas within the boreal caribou distribution (≤ 20%^54^; Supplementary Fig. S1). These seven species were selected because > 50% of their Canadian extent of occurrence was within the boreal caribou distribution, increasing the likelihood that selected species were representative of the boreal forest (i.e. minimizing the inclusion of species at the periphery of their distribution). The unique species are the Whooping Crane (*Gus Americana*), Plains and Wood Bison, Lake Sturgeon, occupying areas of the Northwest Territories, Alberta, British Columbia, Saskatchewan and Manitoba, and the Atlantic Salmon and Barrow’s Goldeneye in Québec and Labrador (Fig. 2c; Supplemental Data S4). We defined indicator-specific hotspots as cells in the top quartile of Canada-wide values of species richness and taxonomic diversity and unique species hotspots as cells occupied by any of the seven unique species^55^.

### Climate Refugia

We used the existing Adaptwest Climate Adaptation Project ecoregional refugia^56^ to define climate refugia, or areas expected to experience minimal change between present and future climates. The 1 km^2^ gridded dataset provides an index of climate-change refugia potential for each North American ecoregion by calculating the distance in km between current and future species distribution models for each cell^17^. Each cell value is adjusted using a dispersal function to down-weight rare, long distance dispersal events (i.e. lower probability of colonizing sites at larger distances). The estimated backwards climate velocity values range between 0 and 1, with a value of 1 indicating overlap or close proximity between future and current climates. We chose the Representative Concentration Pathways (RCP) 8.5 projections for our analysis. RCP 8.5 projections forecast greenhouse gas emissions with little to no mitigation actions^57^ and, as such, represent the uppermost extreme in terms of future climate change scenarios (Supplemental Data S5). Other studies have shown that more optimistic RCP projections (e.g. RCP 4.5) produce similar spatial pattern of climate refugia despite an overall increase in backward climate velocities values^17^.We used shorter-term projections (i.e., 2050 instead of 2080) because of the lesser uncertainty in modelled outputs compared to longer-term projections.

We used a dispersal-based definition for climate refugia or climate hotspots building on the notion that dispersal would affect a species’ ability to respond to climatic shifts in distribution^15^. We summarized major reviews from the published literature on dispersal for a wide range of taxa representative of those in our analysis. We chose a dispersal rate of 100 km/century as a reasonable representation for the taxa included in our analysis^58,59^. While this dispersal estimate may be conservative for birds, it likely exceeds the upper dispersal limit for some species (e.g. tree dispersal^60–62^). We used the dispersal function in Stralberg et al.^15^ that models decreases in the backwards climate velocity values as a function of changing rates of dispersal to determine the backwards climate velocity value corresponding to 100 km/century. All cell values ≥  ~ 0.0313 were considered climate refugia or climate hotspots for our boreal species. In short, we defined climate refugia as all climatically constant areas accessible to species dispersing 100 km/century or less.

### Soil carbon

Soil carbon remains relatively stable to a 1 m depth and provides a good representation of total ecosystem C across forest maturity and disturbance regimes, but varies, for example, with soil type and drainage conditions^37^. Cryosols are often ranked second in terms of total carbon content at 1 m depths compared to soils from wetland ecosystems. DeLuca & Boisvenue^37^ and Ping et al. ^63^ reported that very poorly drained cryosols had the highest total 1-m depth carbon content at 608 tonnes/ha across 52 soil types found in Black Spruce (*Picea marina*) dominated forests of Alaska. Accordingly, we used the threshold of ≥ 608 tonnes/ha of total carbon to identify carbon hotspots within the boreal caribou distribution. Soil carbon values to a 1 m depth were extracted from the SOILGRIDS database^64^, for which 608 tonnes/ha represents approximately the 75% percentile of the distribution of soil carbon content in the North American Boreal^37^. The accuracy of SOILGRIDS is similar to other soil databases, ranging between 20 and 50%; however, the SOILGRIDS database is globally consistent and available at a fine resolution. The 250 m raster soil carbon estimates were generalized to the 1 km climate refugia index grids (Supplemental Data S6).

### Hotspots within Canada’s protected areas network

We used the Canadian Protected and Conserved Areas Database^36^ to identify existing areas used in Canada’s international reporting on progress towards achieving its commitments under the UNCBD. Canada reports on protected areas categories I through VI, assessed according to International Union for Conservation of Nature standards (see Supplemental Table S2). The protected and conserved areas database was cropped to the boreal caribou distribution (Supplemental Data S7).

We used a gap analysis^35^ to assess whether the representation of hotspots within the protected areas network across the boreal caribou distribution was consistent with their availability. A gap analysis allows for the identification of elements that are poorly represented in a conservation network by comparing the network’s current state to an expected or desired state^35^. We defined the current state of the protected areas network in terms of the total area for each of the biological diversity, climate refugia and carbon hotspots. We defined the expected state as that found across the distribution of boreal caribou using random sampling without replacement. From the total area of 2, 440, 837 cells, we selected 100 random samples of 254, 646 cells without replacement (each the size of the protected areas network). We estimated the expected value from the sample, with 95% confidence interval, of the area within each hotspot class.

We examined the degree of spatial overlap among hotspots outside the protected areas networks. Areas of high overlap may be good candidates for future expansions of the protected areas network, allowing Canada to achieve multiple conservation objectives simultaneously. We calculated the centered version of the Jaccard/Tanimoto similarity coefficient^65^ to assess whether the occurrence of all paired hotspots across the boreal caribou distribution were independent (pairwise comparisons, N = 10). Unlike the conventional Jaccard/Tanimoto index, the centered value represents overlap between two data sets (hotspots here) as a probability (as opposed to a ratio) by accounting for differences in hotspot prevalences^65^. Positive coefficients indicate that overlap between hotspots is greater than expected based on occurrence, negative values less overlap than expected, and zero little to no overlap. All coefficients were generated in R version 3.6.3 using the ‘jaccard’ package^66^.

## Supplementary Information

Supplementary Information.

## Data Availability

Data are available using the following link https://figshare.com/projects/Protecting_boreal_caribou_habitat_can_help_conserve_biodiversity_and_safeguard_large_quantities_of_soil_carbon_in_Canada/137448. Links to the individual datasets are available in Supplemental Information.

## References

[1] Barnosky AD (2011). Has the Earth’s sixth mass extinction already arrived?. Nature.

[2] Ceballos G (2015). Accelerated human-induced species losses: Entering the sixth mass extinction. Sci. Adv..

[3] Purvis A (2019). Nature.

[4] Balvernara P (2019). Drivers. Change.

[5] Carrol C, Noss RF (2020). Rewilding in the face of climate change. Conserv. Biol..

[6] Barr SL, Larson BMH, Beechey TJ, Scott DJ (2020). Assessing climate change adaptation progress in Canada’s protected areas. Can. Geog..

[7] Convention on Biological Diversity. Aichi Target 11, Convention on Biological Diversity. https://www.cbd.int/aichi-targets/target/11. Accessed 14 May 2021.

[8] United Nations. Climate Change Pathways. https://unfccc.int/climate-action/marrakech-partnership/reporting-and-tracking/climate_action_pathways. Accessed 12 Sept 2022.

[9] Government of Canada. Canada’s nature legacy: Protecting our nature conservation/nature-legacy.html (2021).

[10] Coristine LE (2017). Informing Canada’s commitment to biodiversity conservation: A science-based framework to help guide protected areas designation through Target 1 and beyond. Facets.

[11] De Barros AE (2013). Identification of areas in Brazil that optimize areas that optimize conservation of forest carbon, Jaguars and Biodiversity. Conserv. Biol..

[12] Jantz P, Scott S, Laporte N (2014). Carbon stock corridors to mitigate climate change and promote biodiversity in the tropics. Nat. Clim. Change.

[13] Beaudrot L (2016). Limited carbon and biodiversity co-benefits for tropical mammals and birds. Ecol. Appl..

[14] Morelli TL (2020). Climate-change refugia: Biodiversity in a slow lane. Front. Ecol. Environ..

[15] Stralberg (2018). Macrorefugia for North American trees ad songbirds: Climatic limiting factors and multi-scale topographic influences. Glob. Ecol. Biogeogr..

[16] Caroll C, Ray JC (2020). Maximizing the effectiveness of national commitments to protected area expansion for conserving biodiversity and ecosystem carbon under climate change. Glob. Chang Biol..

[17] Bradshaw CJ, Warkentin IG, Sodhi NS (2009). Urgent preservation of boreal carbon stocks and biodiversity. Trends Ecol. Evol..

[18] Harris LI (2022). The essential carbon service provided by northern peatlands. Front. Ecol. Environ..

[19] Environment and Climate Change Canada. Canadian Environmental Sustainability Indicators: Canada's conserved areas. environmental-indicators/conserved-areas.html (2020).

[20] Office of the Auditor General of Canada. Lessen learnt from 30 years of climate change challenges and opportunities. https://www.oag-bvg.gc.ca/internet/English/att__e_43948.html#hd3l (2020).

[21] Shea, T. *et al.* Canada’s Conservation Vision: A report of the National Advisory Panel. Government of Canada, 43 pp (2018).

[22] Environment and Climate Change Canada. Pan-Canadian Approach to transforming species at risk conservation in Canada. species-at-risk-conservation.html (2018).

[23] Bergerund AT (1988). Caribou, wolves and man. Trends Ecol. Evol..

[24] Vernier LA (2014). Effects of natural resource development on the terrestrial biodiversity of Canadian boreal forests. Environ. Rev..

[25] Wells JV, Dawson N, Culver N, Reid FA, Slegers SM (2020). The state of conservation in North America’s Borel Forest: Issues and opportunities. Front. For. Glob. Change.

[26] COSEWIC. COSEWIC assessment and update status report on the woodland caribou *Rangifer tarandus caribou* in Canada. Committee on the Status of Endangered Wildlife in Canada. Ottawa. xi + 98 pp. (2002).

[27] COSEWIC. COSEWIC assessment and status report on the caribou *Rangifer tarandus*, Newfoundland population, Atlantic-Gaspésie population and Boreal population, in Canada. Committee on the Status of Endangered Wildlifein Canada. Ottawa. xxiii + 128 pp. (2014).

[28] Environment and Climate Change Canada. Amended Recovery Strategy for the Woodland Caribou (*Rangifer tarandus caribou*), Boreal Population, in Canada. *Species at Risk Act* Recovery Strategy Series. Environment and Climate Change Canada, Ottawa. xiii + 143pp. (2020).

[29] Environment and Climate Change Canada. Report on the Progress of Recovery Strategy Implementation for the Woodland Caribou (Rangifer tarandus caribou), Boreal population in Canada for the Period 2012–2017. Species at Risk Act Recovery Strategy Series. Environment and Climate Change Canada, Ottawa. ix + 94 (2017).

[30] Hebblewhite M (2017). Billion dollar boreal woodland caribou and the biodiversity impacts of the global oil and gas industry. Biol. Conserv..

[31] Fortin D, McLoughlin PD, Hebblewhite M (2020). When the protection of a threatened species depends on the economy of a foreign nation. PLoS ONE.

[32] Drever RC (2019). Conservation through co-occurrence: Woodland caribou as a focal species for boreal biodiversity. Biol. Conserv..

[33] Government of Canada. Pan-Canadian Framework on clean growth and climate change climatechange/pan-canadian-framework.html.

[34] Bradshaw CJ, Warkentin IG (2015). Global estimates of boreal forest carbon stocks and flux. Glob. Planet Chang.

[35] Jennings MD (2010). Gap analysis: Concept, methods, recent results. Land Ecol..

[36] Environment and Climate Change Canada. Canadian Protected and Conserved Areas database. national-wildlife-areas/protected-conserved-areas-database (2019).

[37] DeLuca TH, Boisvenue C (2012). Boreal forest soil carbon: Distribution function and modelling. Forestry.

[38] Price (2013). Anticipating the consequences of climate change for Canada’s boreal forest ecosystems. Environ. Rev..

[39] Southee FM, Edwards BA, Chetkiewicz CB, O’Connor CM (2021). Freshwater conservation planning in the far north of Ontario, Canada: Identifying priority watersheds for conservation of fish biodiversity in an intact boreal landscape. Facets.

[40] Mitchell MGE (2021). Identifying key ecosystem service providing areas to inform national-scale conservation planning. Environ. Res. Lett..

[41] Labadie GPD, McLoughlin MH, Fortin D (2021). Insect-mediated apparent competition between mammals in a boreal food web. Proc. Natl. Acad. Sci. U S A..

[42] Cameron V, Hargreaves AL (2020). Spatial distribution and conservation hotspots of mammals in Canada. Facets.

[43] Ceballos G, Ehrlich PR (2016). Global mammal distributions, biodiversity hotspots, and conservation. PNAS.

[44] Anielski, M. & Wilson, S. Counting Canada’s natural capital: Assessing the real value of Canada’s boreal ecosystems. Ottawa, On: Canadian Boreal Initiative and Pembina Institute counting-canadas-natural-capital (2009).

[45] Kumaraswamy S, Udyakumar M (2011). Biodiversity banking: A strategic conservation mechanism. Biodiver. Conserv..

[46] Garnett ST (2018). A spatial overview of the global importance of Indigenous lands for conservation. Nat. Sustain..

[47] Godden L, Cowell S (2016). Conservation planning and Indigenous governance in Australia's Indigenous Protected Areas. Restor. Ecol..

[48] Greg Brown B, Fagerholm N (2021). Empirical PPGIS/PGIS mapping of ecosystem services: A review and evaluation. Ecol. Ser..

[49] Martin AE, Neave E, Kirby P, Drever CR, Johnson CA (2022). Multi-objective optimization can balance trade-offs among boreal caribou, biodiversity, and climate change objectives when conservation hotspots do not overlap. Sci. Rep..

[50] COSEWIC. Canadian Wildlife Species at Risk. Committee on the Status of Endangered Wildlife in Canada (2018).

[51] Alberta Environment and Parks and Alberta Conservation Association. Status of the Arctic Grayling (*Thymallus arcticus*) in Alberta: Update 2015. Alberta Environment and Parks. Alberta Wildlife Status Report No. 57 (Update 2015). Edmonton, AB. 96 pp. (2015).

[52] Environment and Climate Change Canada (ECCC). 2016. Range map extents, species at risk, Canada. Government of Canada. Open Government Dataset. https://open.canada.ca/data/en/dataset/d00f8e8c-40c4-435a-b790-980339ce3121.

[53] Magurran AE (2004). Measuring Biological Diversity.

[54] Caissy P, Klemet-N’Guessan S, Jackiw R, Eckert CG, Hargreaves AL (2020). High conservation priority of range-edge plant populations not matched by habitat protection or research effort. Biol. Conserv..

[55] Gaston KJ (1994). Rarity.

[56] Stralberg D (2019). Zenodo..

[57] Fuss S (2014). Betting on negative emissions. Nat. Clim. Change.

[58] Chen I, Hill JK, Ohlemüller RDB, Thomas CD (2011). Rapid range shifts of species associated with high levels of climate warming. Science.

[59] Woodall CW (2009). An indicator of tree migration in forests of the eastern United States. For. Ecol. Manag..

[60] Iverson LR, Schwartz MW, Prasad AM (2004). How fast and far might tree species migrate in the eastern United States due to climate change?. Glob. Ecol. Biogeogr..

[61] McLachlan JS, Hellmann JJ, Schwartz MW (2007). A framework for debate of assisted migration in an era of climate change. Conserv. Biol..

[62] Sittaro F, Paquette A, Messier C, Nock CA (2017). Tree range expansion in eastern North America fails to keep pace with climate warming at northern range limits. Glob. Change Biol..

[63] Ping CL (2010). Carbon stores and biogeochemical properties of soils under black spruce forest, Alaska. Soil Sci. Soc. Am. J..

[64] Hengl T (2017). SoilGrids250m: Global soil information based on machine learning. PLoS ONE.

[65] Chung NC, Miasojedow B, Startek M, Gambin A (2019). Jaccard/Tanimoto similarity test and estimation methods for biological presence-absence data. BMC Bioinform..

[66] Chung, N. C., Miasojedow, B., Startek, M. & Gambin A. Jaccard: Test Similarity Between Binary Data using Jaccard/Tanimoto Coefficients. R package version 0.1.0. https://CRAN.R-project.org/package=jaccard (2018).

