# Supplementary material for: Protecting boreal caribou habitat can help conserve biodiversity and safeguard large quantities of soil carbon in Canada
Source: Sci Rep. 2022 Oct 12;12:17067. doi: 10.1038/s41598-022-21476-x (PMC9556649; doi:10.1038/s41598-022-21476-x)

Johnson, C. A., Drever, R., Kirby, P. Neave, E. & Martin, A. E. Protecting boreal caribou habitat can help conserve biodiversity and safeguard large quantities of soil carbon in Canada

Supplement Data S1. Shapefiles for species’ distribution <https://figshare.com/articles/dataset/Prepared_species_distributions/19660614>

Supplemental Data S2. Species richness within the boreal caribou distribution <https://figshare.com/articles/dataset/Species_richness_within_the_boreal_caribou_distribution/19640037>

Supplemental Data S3. Taxonomic diversity within the boreal caribou distribution <https://figshare.com/articles/dataset/Taxonomic_diversity_within_the_boreal_caribou_distribution/19640043>

Supplemental Data S4. Unique species within the boreal caribou distribution <https://figshare.com/articles/dataset/Unique_species_within_the_boreal_caribou_distribution/19651767>

Supplemental Data S5. Refugia within the boreal caribou distribution (modified from source) <https://figshare.com/articles/dataset/Refugia_within_the_boreal_caribou_distribution_modified_from_source_/19638771>

Supplemental Data S6. Soil carbon within boreal caribou distribution (modified from source) <https://figshare.com/articles/dataset/Soil_carbon_within_boreal_caribou_distribution_modified_from_source_/19636749>

Supplemental Data S7. Aichi Target 11 protected and conserved areas within the boreal caribou distribution (modified from source) https://figshare.com/articles/dataset/Aichi_Target_11_protected_and_conserved_areas_within_the_boreal_caribou_distribution_modified_from_source_/19651962

Supplemental Table S1. List of species at risk across the boreal caribou distribution. Only species that were threatened by human disturbance were included in the hotspots analyses.

| **Scientific Name** | **Common Name** | **Taxon** | **COSEWIC Status** | **Threatened by human disturbance** | | **Source for distribution mapping** |
| --- | --- | --- | --- | --- | --- | --- |
|  |  |  |  | **YES/NO?** | **Reference** | Reference |
| *Ambystoma mavortium* | Western Tiger Salamander (Prairie / Boreal population) | Amphibians | Special Concern | YES | COSEWIC 2012a | ECCC 2016 |
| *Anaxyrus boreas* | Western Toad (calling population) | Amphibians | Special Concern | YES | COSEWIC 2012b | ECCC 2016 |
| *Anaxyrus boreas* | Western Toad (non-calling population) | Amphibians | Special Concern | YES | COSEWIC 2012b | ECCC 2016 |
| *Lithobates pipiens* | Northern Leopard Frog (Western Boreal/ Prairie populations) | Amphibians | Special Concern | YES | COSEWIC 2009a | ECCC 2016 |
| *Bombus bohemicus* | Gypsy Cuckoo Bumble Bee | Arthropods | Endangered | YES | COSEWIC 2014a | ECCC 2016 |
| *Bombus occidentalis mckayi* | Western Bumble Bee mckayi subspecies | Arthropods | Special Concern | YES | COSEWIC 2014b | ECCC 2016 |
| *Danaus plexippus* | Monarch | Arthropods | Endangered* | YES | COSEWIC 2016a | ECCC 2016 |
| *Epeoloides pilosulus* | Macropis Cuckoo Bee | Arthropods | Endangered | YES | COSEWIC 2011a | ECCC 2016 |
| *Coccinella novemnotata* | Nine-spotted Lady Beetle | Arthropods | Endangered | YES | COSEWIC 2016b | COSEWIC 2016b |
| *Coccinella transversoguttata* | Transverse Lady Beetle | Arthropods | Special Concern | YES | COSEWIC 2016c | COSEWIC 2016c |
| *Bombus terricola* | Yellow-banded Bumble Bee | Arthropods | Special Concern | YES | COSEWIC 2015a | COSEWIC 2015a |
| *Aechmophorus occidentalis* | Western Grebe | Birds | Special Concern | YES | COSEWIC 2014c | ECCC 2016 |
| *Anthus spragueii* | Sprague's Pipit | Birds | Threatened | YES | COSEWIC 2010a | ECCC 2016 |
| *Antrostomus vociferus* | Eastern Whip-poor-will | Birds | Threatened | YES | COSEWIC 2009b | ECCC 2016 |
| *Asio flammeus* | Short-eared Owl | Birds | Special Concern | YES | COSEWIC 2008a | ECCC 2016 |
| *Bucephala islandica* | Barrow's Goldeneye (Eastern population) | Birds | Special Concern | YES | COSEWIC 2000a | ECCC 2016 |
| *Calidris canutus roselaari type* | Red Knot roselaari type | Birds | Threatened | NO | COSEWIC 2007a | ECCC 2016 |
| *Calidris canutus rufa* | Red Knot rufa subspecies | Birds | Endangered | NO | COSEWIC 2007a | ECCC 2016 |
| *Cardellina canadensis* | Canada Warbler | Birds | Threatened | YES | COSEWIC 2008b | ECCC 2016 |
| *Catharus bicknelli* | Bicknell's Thrush | Birds | Threatened | YES | COSEWIC 2009c | ECCC 2016 |
| *Chaetura pelagica* | Chimney Swift | Birds | Threatened | YES | COSEWIC 2018b | ECCC 2016 |
| *Charadrius melodus circumcinctus* | Piping Plover circumcinctus subspecies | Birds | Endangered | YES | COSEWIC 2013a | ECCC 2016 |
| *Chordeiles minor* | Common Nighthawk | Birds | Threatened | YES | COSEWIC 2018c | ECCC 2016 |
| *Contopus cooperi* | Olive-sided Flycatcher | Birds | Special Concern* | YES | COSEWIC 2018d | ECCC 2016 |
| *Contopus virens* | Eastern Wood-pewee | Birds | Special Concern | YES | COSEWIC 2012c | ECCC 2016 |
| *Coturnicops noveboracensis* | Yellow Rail | Birds | Special Concern | YES | COSEWIC 2009d | ECCC 2016 |
| *Dolichonyx oryzivorus* | Bobolink | Birds | Threatened* | YES | COSEWIC 2010b | ECCC 2016 |
| *Euphagus carolinus* | Rusty Blackbird | Birds | Special Concern | YES | COSEWIC 2017a | ECCC 2016 |
| *Grus americana* | Whooping Crane | Birds | Endangered | YES | COSEWIC 2010c | ECCC 2016 |
| *Hirundo rustica* | Barn Swallow | Birds | Threatened | YES | COSEWIC 2011b | ECCC 2016 |
| *Histrionicus histrionicus* | Harlequin Duck (Eastern population) | Birds | Special Concern | YES | COSEWIC 2013b | ECCC 2016 |
| *Hylocichla mustelina* | Wood Thrush | Birds | Threatened | YES | COSEWIC 2012d | ECCC 2016 |
| *Ixobrychus exilis* | Least Bittern | Birds | Threatened | YES | COSEWIC 2009e | ECCC 2016 |
| *Lanius ludovicianus excubitorides* | Loggerhead Shrike Prairie subspecies | Birds | Threatened | YES | COSEWIC 2004 | ECCC 2016 |
| *Melanerpes erythrocephalus* | Red-headed Woodpecker | Birds | Endangered* | YES | COSEWIC 2018e | ECCC 2016 |
| *Numenius borealis* | Eskimo Curlew | Birds | Endangered | NO | COSEWIC 2009f | ECCC 2016 |
| *Phalaropus lobatus* | Red-necked Phalarope | Birds | Special Concern | YES | COSEWIC 2014d | ECCC 2016 |
| *Podiceps auritus* | Horned Grebe (Western population) | Birds | Special Concern* | YES | COSEWIC 2009g | ECCC 2016 |
| *Riparia riparia* | Bank Swallow | Birds | Threatened | YES | COSEWIC 2013c | ECCC 2016 |
| *Sturnella magna* | Eastern Meadowlark | Birds | Threatened | YES | COSEWIC 2011c | ECCC 2016 |
| *Tryngites subruficollis* | Buff-breasted Sandpiper | Birds | Special Concern | NO | COSEWIC 2012e | ECCC 2016 |
| *Tympanuchus cupido* | Greater Prairie-Chicken | Birds | Extirpated | NO | COSEWIC 2000b | ECCC 2016 |
| *Coccothraustes vespertinus* | Evening Grosbeak | Birds | Special Concern | YES | COSEWIC 2016d | COSEWIC 2016d |
| *Zonotrichia querula* | Harris's Sparrow | Birds | Special Concern | NO | COSEWIC 2017b | COSEWIC 2017b |
| *Leptogium rivulare* | Flooded Jellyskin | Lichens | Special Concern* | YES | COSEWIC 2015b | ECCC 2016 |
| *Peltigera hydrothyria* | Eastern Waterfan | Lichens | Threatened | YES | COSEWIC 2013d | ECCC 2016 |
| *Quadrula quadrula* | Mapleleaf (Saskatchewan-Nelson Rivers population) | Molluscs | Threatened | YES | COSEWIC 2016e | COSEWIC 2016e |
| *Bison bison athabascae* | Wood Bison | Mammals | Special Concern | YES | COSEWIC 2013e | ECCC 2016 |
| *Bison bison bison* | Plains Bison | Mammals | Threatened | YES | COSEWIC 2013e | COSEWIC 2013e |
| *Canis sp. cf. lycaon* | Eastern Wolf | Mammals | Threatened | YES | COSEWIC 2015c | ECCC 2016 |
| *Gulo gulo* | Wolverine | Mammals | Special Concern | YES | COSEWIC 2014e | ECCC 2016 |
| *Ochotona collaris* | Collared Pika | Mammals | Special Concern | YES | COSEWIC 2011d | ECCC 2016 |
| *Rangifer tarandus* | Caribou (Central Mountain population) | Mammals | Endangered | YES | COSEWIC 2014f | ECCC 2016 |
| *Rangifer tarandus* | Woodland Caribou (Northern Mountain population) | Mammals | Special Concern | YES | COSEWIC 2014f | ECCC 2016 |
| *Rangifer tarandus* | Caribou (Eastern Migratory population) | Mammals | Endangered | YES | COSEWIC 2017c | COSEWIC 2011f |
| *Rangifer tarandus* | Caribou (Barren-ground population) | Mammals | Threatened | YES | COSEWIC 2016f | COSEWIC 2011f |
| *Taxidea taxus taxus* | American Badger taxus subspecies | Mammals | Special Concern* | YES | COSEWIC 2012f | ECCC 2016 |
| *Ursus maritimus* | Polar Bear | Mammals | Special Concern | NO | COSEWIC 2018f | ECCC 2016 |
| *Ursus arctos (Western population)* | Grizzly Bear (Western population) | Mammals | Special Concern* | YES | COSEWIC 2012g | COSEWIC 2012g |
| *Phoca vitulina mellonae* | Harbour Seal | Freshwater Mammal | Endangered | NO | COSEWIC 2018g | ECCC 2016 |
| *Perimyotis subflavus* | Bat, Tri-colored | Mammals | Endangered | YES | COSEWIC 2013f | COSEWIC 2013f |
| *Myotis lucifugus* | Myotis, little brown | Mammals | Endangered | YES | COSEWIC 2013f | COSEWIC 2013f |
| *Myotis, septentrionalis* | Myotis, northern | Mammals | Endangered | YES | COSEWIC 2013f | COSEWIC 2013f |
| *Chelydra serpentina* | Snapping Turtle | Reptiles | Special Concern | YES | COSEWIC 2008c | ECCC 2016 |
| *Chrysemys picta bellii* | Western Painted Turtle (Intermountain - Rocky Mountain population) | Reptiles | Special Concern | YES | COSEWIC 2016g | ECCC 2016 |
| *Chrysemys picta marginalis* | Midland Painted Turtle | Reptiles | Special Concern | YES | COSEWIC 2018h | COSEWIC 2018h |
| *Achillea millefolium var. megacephalum* | Large-headed Woolly Yarrow | Vascular Plants | Special Concern | NO | COSEWIC 2018i | ECCC 2016 |
| *Armeria maritima ssp. interior* | Athabasca Thrift | Vascular Plants | Special Concern | NO | COSEWIC 2018j | ECCC 2016 |
| *Astragalus robbinsii var. fernaldii* | Fernald's Milk-vetch | Vascular Plants | Special Concern | YES | Environment Canada 2011 | ECCC 2016 |
| *Cirsium pitcheri* | Pitcher's Thistle | Vascular Plants | Special Concern | YES | COSEWIC 2010d | ECCC 2016 |
| *Deschampsia mackenzieana* | Mackenzie Hairgrass | Vascular Plants | Special Concern | NO | COSEWIC 2018j | ECCC 2016 |
| *Juglans cinerea* | Butternut | Vascular Plants | Endangered | YES | COSEWIC 2017d | ECCC 2016 |
| *Panax quinquefolius* | American Ginseng | Vascular Plants | Endangered | YES | COSEWIC 2000c | ECCC 2016 |
| *Salix brachycarpa var. psammophila* | Sand-dune Short-capsuled Willow | Vascular Plants | Special Concern | NO | COSEWIC 2018j | ECCC 2016 |
| *Salix silicicola* | Felt-leaf Willow (Blanket-leaved willow) | Vascular Plants | Special Concern | NO | COSEWIC 2018j | ECCC 2016 |
| *Salix turnorii* | Turnor's Willow | Vascular Plants | Special Concern | NO | COSEWIC 2018j | ECCC 2016 |
| *Tanacetum huronense var. floccosum* | Floccose Tansy | Vascular Plants | Special Concern | NO | COSEWIC 2018j | ECCC 2016 |
| *Salvelinus malma malma* | Dolly Varden | Fish | Special Concern | YES | COSEWIC 2010e | COSEWIC 2010e |
| *Ichthyomyzon fossor* | Northern Brook Lamprey (Great Lakes Upper St. Lawrence populations) | Fish | Special Concern | YES | COSEWIC 2007b | COSEWIC 2007b |
| *Myoxocephalus thompsonii* | Deepwater Sculpin (Great Lakes Upper St. Lawrence) | Fish | Special Concern | YES | COSEWIC 2017e | COSEWIC 2017e |
| *Ictiobus cyprinellus* | Bigmouth Buffalo (Saskatchewan Nelson Riverpopulations) | Fish | Special Concern | YES | COSEWIC 2009h | DFO 2017 |
| *Acipenser fulvescens* | Sturgeon, Lake (Western Hudson Bay populations) | Freshwater Fish | Endangered | YES | COSEWIC 2017f | COSEWIC 2017f |
| *Acipenser fulvescens* | Sturgeon, Lake (Saskatchewan-Nelson River populations) | Freshwater Fish | Endangered | YES | COSEWIC 2017f | COSEWIC 2017f |
| *Acipenser fulvescens* | Sturgeon, Lake (Southern Hudson Bay - James Bay populations) | Freshwater Fish | Special Concern | YES | COSEWIC 2017f | COSEWIC 2017f |
| *Acipenser fulvescens* | Sturgeon, Lake (Great Lakes-Upper St. Lawrence populations) | Freshwater Fish | Threatened | YES | COSEWIC 2017f | COSEWIC 2017f |
| *Oncorhynchus mykiss* | Trout, Rainbow (Athabasca River population) | Freshwater Fish | Endangered | YES | COSEWIC 2014g | COSEWIC 2014g |
| *Ichthyomyzon unicuspis* | Lamprey, Silver (Great Lakes-Upper St. Lawrence populations) | Freshwater Fish | Special Concern | YES | COSEWIC 2011e | COSEWIC 2011e |
| *Salmo salar* | Salmon, Atlantic (Quebec Eastern North Shore population) | Freshwater Fish | Special Concern | YES | COSEWIC 2010f | COSEWIC 2010f; DFO and MNRF 2008 |
| *Salmo salar* | Salmon, Atlantic (Quebec Western North Shore population) | Freshwater Fish | Special Concern | YES | COSEWIC 2010^f^ | COSEWIC 2010f; DFO and MNRF 2008 |
| *Salmo salar* | Salmon, Atlantic (Inner St. Lawrence population) | Freshwater Fish | Special Concern | YES | COSEWIC 2010^f^ | COSEWIC 2010f; DFO and MNRF 2008 |
| *Salvelinus confluentus* | Trout, Bull (Western Arctic populations) | Freshwater Fish | Special Concern | YES | COSEWIC 2012^h^ | COSEWIC 2012h |
| *Coregonus zenithicus* | Cisco, Shortjaw | Freshwater Fish | Threatened | YES | COSEWIC 2003 | COSEWIC 2003 |
| *Anguilla rostrata* | American Eel | Freshwater Fish | Threatened | YES | COSEWIC 2012i | COSEWIC 2012i |
| *Prosopium coulterii* | Whitefish, Pygmy (Great Lakes - Upper St. Lawrence populations) | Freshwater Fish | Threatened | YES | COSEWIC 2016h | COSEWIC 2016h  Lake Superior shoreline buffered to 4km to account for seasonal spawning migration |
| *Thymallus arcticus* | Arctic Grayling | Freshwater Fish | Unclassified (COSEWIC 2018a), Special Concern (AB) | YES | Alberta Environment and Parks and Alberta Conservation Association 2015 | Alberta Environment and Parks and Alberta Conservation Association 2015 |

**^*^**Status from ECCC 2016 database updated based on COSEWIC 2018a

References

Alberta Environment and Parks and Alberta Conservation Association. 2015. Status of the

Arctic Grayling (*Thymallus arcticus*) in Alberta: Update 2015. Alberta Environment and Parks.

Alberta Wildlife Status Report No. 57 (Update 2015). Edmonton, AB. 96 pp. <https://www.ab-conservation.com/downloads/AWSR/Reptile%20Amphibian%20and%20Fish%20Reports/Status-of-Arctic-Grayling-in-Alberta-Update-2015_2017.pdf>

COSEWIC. 2018a. Canadian Wildlife Species at Risk. Committee on the Status of Endangered Wildlife in Canada. <https://wildlife-species.canada.ca/species-risk-registry/virtual_sara/files/species/Cl-csar2018-v00-2019Feb1-Eng.pdf>

COSEWIC. 2018b. COSEWIC assessment and status report on the Chimney Swift *Chaetura pelagica* in Canada. Committee on the Status of Endangered Wildlife in Canada. Ottawa. xii + 63 pp. <http://www.registrelep-sararegistry.gc.ca/default.asp?lang=en&n=24F7211B-1>). <https://wildlife-species.canada.ca/species-risk-registry/virtual_sara/files/cosewic/srChimneySwift2018e.pdf>

COSEWIC. 2018c. COSEWIC assessment and status report on the Common Nighthawk *Chordeiles minor* in Canada. Committee on the Status of Endangered Wildlife in Canada. Ottawa. xi + 50 pp. <https://wildlife-species.canada.ca/species-risk-registry/virtual_sara/files/cosewic/srCommonNighthawk2018e.pdf>

COSEWIC. 2018d. COSEWIC assessment and status report on the Olive-sided Flycatcher *Contopus cooperi* in Canada. Committee on the Status of Endangered Wildlife in Canada. Ottawa. ix + 52 pp. <https://wildlife-species.canada.ca/species-risk-registry/virtual_sara/files/cosewic/srOlive-sidedFlycatcher2018e.pdf>

COSEWIC. 2018e. COSEWIC assessment and status report on the Red-headed Woodpecker *Melanerpes erythrocephalus* in Canada. Committee on the Status of Endangered Wildlife in Canada. Ottawa. xii + 60 pp. <https://wildlife-species.canada.ca/species-risk-registry/virtual_sara/files/cosewic/srRed-headedWoodpecker2018e.pdf>

COSEWIC. 2018f. COSEWIC assessment and status report on the Polar Bear *Ursus maritimus* in Canada. Committee on the Status of Endangered Wildlife in Canada. Ottawa. xv + 113 pp. <https://wildlife-species.canada.ca/species-risk-registry/virtual_sara/files/cosewic/OursBlancPolarBear-2019-Eng.pdf>

COSEWIC. 2018g. COSEWIC status appraisal summary on the Harbour Seal Lacs des Loups Marins subspecies *Phoca vitulina mellonae* in Canada. Committee on the Status of Endangered Wildlife in Canada. Ottawa. xiv pp. <https://wildlife-species.canada.ca/species-risk-registry/virtual_sara/files/cosewic/Sas-PhoqueCommunHarbourSealLacsLoupsMarins-v00-2018Dec-Eng.pdf>

COSEWIC. 2018h. COSEWIC assessment and status report on the Midland Painted Turtle *Chrysemys picta marginata* and the Eastern Painted Turtle *Chrysemys picta picta* in Canada. Committee on the Status of Endangered Wildlife in Canada. Ottawa. xvi + 107 pp. <https://wildlife-species.canada.ca/species-risk-registry/virtual_sara/files/cosewic/srMidlandPaintedTurtleEasternPaintedTurtle2018e.pdf>

COSEWIC. 2018i. COSEWIC assessment and status report on the Athabasca Endemics, Large-headed Woolly Yarrow (*Achillea millefolium var. megacephala*), Athabasca Thrift (Armeria maritima ssp. interior), Mackenzie Hairgrass (*Deschampsia mackenzieana*), Sand-dune Short-capsuled Willow (*Salix brachycarpa* var. *psammophila*), Turnor’s Willow (*Salix turnorii*), Blanket-leaved Willow (*Salix silicicola*), and Floccose Tansy (*Tanacetum huronense* var. *floccosum*) in Canada. Committee on the Status of Endangered Wildlife in Canada. Ottawa. xlvii + 83 pp. <https://wildlife-species.canada.ca/species-risk-registry/virtual_sara/files/cosewic/Sr-EndemiquesAthabascaEndemics-v00-2018Dec-Eng.pdf>

COSEWIC. 2017a. COSEWIC assessment and status report on the Rusty Blackbird *Euphagus carolinus* in Canada. Committee on the Status of Endangered Wildlife in Canada. Ottawa. xi + 64 pp. <https://wildlife-species.canada.ca/species-risk-registry/virtual_sara/files/cosewic/sr_Rusty%20Blackbird_2017_e.pdf>

COSEWIC. 2017b. COSEWIC assessment and status report on the Harris’s Sparrow *Zonotrichia querula* in Canada. Committee on the Status of Endangered Wildlife in Canada. Ottawa. x + 36 pp. <https://wildlife-species.canada.ca/species-risk-registry/virtual_sara/files/cosewic/sr_Harris's%20Sparrow_2017_e.pdf>

COSEWIC. 2017c. COSEWIC assessment and status report on the Caribou *Rangifer tarandus*, Eastern Migratory population and Torngat Mountains population, in Canada. Committee on the Status of Endangered Wildlife in Canada. Ottawa. xvii + 68 pp. <https://wildlife-species.canada.ca/species-risk-registry/virtual_sara/files/cosewic/sr_Caribou%20Eastern%20Migratory%20Torngat%20Mountains%20populations_2017_e.pdf>

COSEWIC. 2017d. COSEWIC assessment and status report on the Butternut *Juglans cinerea* in Canada. Committee on the Status of Endangered Wildlife in Canada. Ottawa. xiii + 74 pp. <https://wildlife-species.canada.ca/species-risk-registry/virtual_sara/files/cosewic/sr_Butternut_2017_e.pdf>

COSEWIC. 2017e. COSEWIC assessment and status report on the Deepwater Sculpin *Myoxocephalus thompsonii*, Great Lakes-Upper St. Lawrence populations, Southern Hudson Bay-James Bay populations, Saskatchewan-Nelson River populations, Waterton Lake population, Western Hudson Bay populations and Western Arctic populations in Canada. Committee on the Status of Endangered Wildlife in Canada. Ottawa. xxxvii + 61 pp. <https://wildlife-species.canada.ca/species-risk-registry/virtual_sara/files/cosewic/sr_Deepwater%20Sculpin_2017_e.pdf>

COSEWIC. 2017f. COSEWIC assessment and status report on the Lake Sturgeon *Acipenser fulvescens*, Western Hudson Bay populations, Saskatchewan-Nelson River populations, Southern Hudson Bay-James Bay populations and Great Lakes-Upper St. Lawrence populations in Canada. Committee on the Status of Endangered Wildlife in Canada. Ottawa. xxx + 153 pp. <https://wildlife-species.canada.ca/species-risk-registry/virtual_sara/files/cosewic/sr_Lake%20Sturgeon_2017_e.pdf>

COSEWIC. 2016a. COSEWIC assessment and status report on the Monarch *Danaus plexippus* in Canada. Committee on the Status of Endangered Wildlife in Canada. Ottawa. xiii + 59 pp.

<https://wildlife-species.canada.ca/species-risk-registry/virtual_sara/files/cosewic/sr_Monarch_2016_e.pdf>

COSEWIC. 2016b. COSEWIC assessment and status report on the Nine-spotted Lady Beetle *Coccinella novemnotata* in Canada. Committee on the Status of Endangered Wildlife in Canada. Ottawa. x + 57 pp. (<http://www.registrelep-sararegistry.gc.ca/default_e.cfm>). <https://species-registry.canada.ca/index-en.html#/documents/3048>

COSEWIC. 2016c. COSEWIC assessment and status report on the Transverse Lady Beetle *Coccinella transversoguttata* in Canada. Committee on the Status of Endangered Wildlife in Canada. Ottawa. xi + 57 pp.
<https://wildlife-species.canada.ca/species-risk-registry/virtual_sara/files/cosewic/sr_Transverse%20Lady%20Beetle_2016_e.pdf>

COSEWIC. 2016d. COSEWIC assessment and status report on the Evening Grosbeak *Coccothraustes vespertinus* in Canada. Committee on the Status of Endangered Wildlife in Canada. Ottawa. xi + 64 pp. <https://wildlife-species.canada.ca/species-risk-registry/virtual_sara/files/cosewic/sr_Evening%20Grosbeak_2016_e.pdf>

COSEWIC. 2016e. COSEWIC assessment and status report on the Mapleleaf *Quadrula quadrula*, Great Lakes - Upper St. Lawrence population and Saskatchewan - Nelson Rivers population, in Canada. Committee on the Status of Endangered Wildlife in Canada. Ottawa. xi + 86 pp. <https://wildlife-species.canada.ca/species-risk-registry/virtual_sara/files/cosewic/sr_Mapleleaf_2016_e.pdf>

COSEWIC. 2016f. COSEWIC assessment and status report on the Caribou *Rangifer tarandus*, Barren-ground population, in Canada. Committee on the Status of Endangered Wildlife in Canada. Ottawa. xiii + 123 pp. <https://wildlife-species.canada.ca/species-risk-registry/virtual_sara/files/cosewic/sr_Caribou%20Barren-ground_2016_e.pdf>

COSEWIC. 2016g. COSEWIC assessment and status report on the Western Painted Turtle *Chrysemys picta bellii*, Pacific Coast population, Intermountain – Rocky Mountain population and Prairie/Western Boreal – Canadian Shield population, in Canada. Committee on the Status of Endangered Wildlife in Canada. Ottawa. xxi + 95 pp. <https://wildlife-species.canada.ca/species-risk-registry/virtual_sara/files/cosewic/sr_Western%20Painted%20Turtle_2016_e.pdf>

COSEWIC. 2016h. COSEWIC assessment and status report on the Pygmy Whitefish *Prosopium coulterii*, Southwestern Yukon Beringian populations, Yukon River populations, Pacific populations, Western Arctic populations, Great Lakes – Upper St. Lawrence populations, Waterton Lake populations and Saskatchewan - Nelson Rivers populations in Canada. Committee on the Status of Endangered Wildlife in Canada. Ottawa. iv + 69 pp. <https://wildlife-species.canada.ca/species-risk-registry/virtual_sara/files/cosewic/sr_Pygmy%20Whitefish_2016_e.pdf>

COSEWIC. 2015a. COSEWIC assessment and status report on the Yellow-banded Bumble Bee *Bombus terricola* in Canada. Committee on the Status of Endangered Wildlife in Canada. Ottawa. ix + 60 pp. <https://species-registry.canada.ca/index-en.html#/documents/2775>

COSEWIC. 2015b. COSEWIC assessment and status report on the Flooded Jellyskin *Leptogium rivulare* in Canada. Committee on the Status of Endangered Wildlife in Canada. Ottawa. xii + 48 pp. <https://wildlife-species.canada.ca/species-risk-registry/virtual_sara/files/cosewic/sr_Flooded%20Jellyskin_2015_e.pdf>

COSEWIC. 2015c. COSEWIC assessment and status report on the Eastern Wolf Canis sp. cf. lycaon in Canada. Committee on the Status of Endangered Wildlife in Canada. Ottawa. xii + 67 pp. <https://wildlife-species.canada.ca/species-risk-registry/virtual_sara/files/cosewic/sr_Eastern%20Wolf_2015_e.pdf>

COSEWIC. 2014a. COSEWIC assessment and status report on the Gypsy Cuckoo Bumble *Bombus bohemicus* in Canada. Committee on the Status of Endangered Wildlife in Canada. Ottawa. ix + 56 pp. <https://wildlife-species.canada.ca/species-risk-registry/virtual_sara/files/cosewic/sr_Gypsy%20Cuckoo%20Bumble%20Bee_2014_e.pdf>

COSEWIC. 2014b. COSEWIC assessment and status report on the Western Bumble Bee *Bombus occidentalis*, *occidentalis* subspecies (*Bombus occidentalis occidentalis*) and the *mckayi* subspecies (*Bombus occidentalis mckayi*) in Canada. Committee on the Status of Endangered Wildlife in Canada. Ottawa. xii + 52 pp. <https://wildlife-species.canada.ca/species-risk-registry/virtual_sara/files/cosewic/sr_Western%20Bumble%20Bee_2014_e.pdf>

COSEWIC. 2014c. COSEWIC assessment and status report on the Western Grebe *Aechmophorus occidentalis* in Canada. Committee on the Status of Endangered Wildlife in Canada. Ottawa. x + 55 pp. <https://wildlife-species.canada.ca/species-risk-registry/virtual_sara/files/cosewic/sr_Western%20Grebe_2014_e%20.pdf>

COSEWIC. 2014d. COSEWIC assessment and status report on the Red-necked Phalarope *Phalaropus lobatusin* Canada. Committee on the Status of Endangered Wildlife in Canada. Ottawa. x + 52 pp.(www.registrelep-sararegistry.gc.ca/default_e.cfm). <https://wildlife-species.canada.ca/species-risk-registry/virtual_sara/files/cosewic/sr_Red-necked%20Phalarope_2014_e.pdf>

COSEWIC. 2014e. COSEWIC assessment and status report on the Wolverine *Gulo gulo* in Canada. Committee on the Status of Endangered Wildlife in Canada. Ottawa. xi + 76 pp. <https://wildlife-species.canada.ca/species-risk-registry/virtual_sara/files/cosewic/sr_Wolverine_2014_e.pdf>

COSEWIC. 2014f. COSEWIC assessment and status report on the Caribou *Rangifer tarandus*, Northern Mountain population, Central Mountain population and Southern Mountain population in Canada. Committee on the Status of Endangered Wildlife in Canada. Ottawa. xxii + 113 pp. <https://wildlife-species.canada.ca/species-risk-registry/virtual_sara/files/cosewic/sr_Caribou_Northern_Central_Southern_2014_e.pdf>

COSEWIC. 2014g. COSEWIC assessment and status report on the Rainbow Trout *Oncorhynchus mykissin* Canada. Committee on the Status of Endangered Wildlife in Canada. Ottawa. xi + 60 pp. <https://wildlife-species.canada.ca/species-risk-registry/virtual_sara/files/cosewic/sr_Rainbow%20Trout_2014_e.pdf>

COSEWIC. 2013a. COSEWIC assessment and status report on the Piping Plover *circumcinctus* subspecies (*Charadrius meloduscircumcinctus*) and the *melodus* subspecies (*Charadrius melodus melodus*) in Canada. Committee on the Status of Endangered Wildlife in Canada. Ottawa. xiv + 39 pp. <https://wildlife-species.canada.ca/species-risk-registry/virtual_sara/files/cosewic/sr_Piping%20Plover_2013_e.pdf>

COSEWIC. 2013b. COSEWIC assessment and status report on the Harlequin Duck *Histrionicus histrionicus* Eastern population in Canada. Committee on the Status of Endangered Wildlife in Canada. Ottawa. ix + 38 pp. <https://wildlife-species.canada.ca/species-risk-registry/virtual_sara/files/cosewic/sr_Harlequin%20Duck_2013_e.pdf>

COSEWIC. 2013c. COSEWIC assessment and status report on the Bank Swallow *Riparia riparia* in Canada. Committee on the Status of Endangered Wildlife in Canada. Ottawa. ix + 48 pp. <https://wildlife-species.canada.ca/species-risk-registry/virtual_sara/files/cosewic/sr_hirondelle_rivage_bank_swallow_1213_e.pdf>

COSEWIC. 2013d. COSEWIC assessment and status report on the Eastern Waterfan *Peltigera hydrothyria* in Canada. Committee on the Status of Endangered Wildlife in Canada. Ottawa. xi + 46 pp. <https://wildlife-species.canada.ca/species-risk-registry/virtual_sara/files/cosewic/sr_Eastern%20Waterfan_2013_e.pdf>

COSEWIC. 2013e. COSEWIC assessment and status report on the Plains Bison *Bison bison bison* and the Wood Bison *Bison bison athabascae* in Canada. Committee on the Status of Endangered Wildlife in Canada. Ottawa. xv + 109 pp.

<https://wildlife-species.canada.ca/species-risk-registry/virtual_sara/files/cosewic/sr_Plains%20Bison%20and%20Wood%20Bison_2013_e.pdf>

COSEWIC. 2013f. COSEWIC assessment and status report on the Little Brown *Myotis Myotis lucifugus*, Northern *Myotis Myotis septentrionalis* and Tri-colored Bat *Perimyotis subflavus* in Canada. Committee on the Status of Endangered Wildlife in Canada. Ottawa. xxiv + 93 pp. <https://wildlife-species.canada.ca/species-risk-registry/virtual_sara/files/cosewic/sr_Little%20Brown%20Myotis&Northern%20Myotis&Tri-colored%20Bat_2013_e.pdf>

COSEWIC. 2012a. COSEWIC assessment and status report on the Western Tiger Salamander *Ambystoma mavortium* in Canada. Committee on the Status of Endangered Wildlife in Canada. Ottawa. xv + 63 pp. <https://wildlife-species.canada.ca/species-risk-registry/virtual_sara/files/cosewic/sr_w_tiger_salaman_tigree_1113_e.pdf>

COSEWIC. 2012b. COSEWIC assessment and status report on the Western Toad *Anaxyrus boreas* in Canada. Committee on the Status of Endangered Wildlife in Canada. Ottawa. xiv + 71 pp. <https://wildlife-species.canada.ca/species-risk-registry/virtual_sara/files/cosewic/sr_Western%20Toad%20_2013_e.pdf>

COSEWIC. 2012c. COSEWIC assessment and status report on the Eastern Wood-pewee *Contopus virens* in Canada. Committee on the Status of Endangered Wildlife in Canada. Ottawa. x + 39 pp. <https://wildlife-species.canada.ca/species-risk-registry/virtual_sara/files/cosewic/sr_Eastern%20Wood-pewee_2013_e.pdf>

COSEWIC. 2012d. COSEWIC assessment and status report on the Wood Thrush *Hylocichla mustelina* in Canada. Committee on the Status of Endangered Wildlife in Canada. Ottawa. ix + 46 pp. <https://wildlife-species.canada.ca/species-risk-registry/virtual_sara/files/cosewic/sr_Wood%20Thrush_2013_e.pdf>

COSEWIC. 2012e. COSEWIC assessment and status report on the Buff-breasted Sandpiper *Tryngites subruficollis* in Canada. Committee on the Status of Endangered Wildlife in Canada. Ottawa. x + 44 pp. <https://wildlife-species.canada.ca/species-risk-registry/virtual_sara/files/cosewic/sr_becasseau_roussatre_buffbreasted_sandpiper_1012_e.pdf>

COSEWIC. 2012f. COSEWIC assessment and status report on the American Badger *Taxidea taxus* in Canada. Committee on the Status of Endangered Wildlife in Canada. Ottawa. iv + 63 pp. <https://wildlife-species.canada.ca/species-risk-registry/virtual_sara/files/cosewic/sr_blaireau_am_badger_1113_e.pdf>

COSEWIC. 2012g. COSEWIC assessment and status report on the Grizzly Bear *Ursus arctos* in Canada. Committee on the Status of Endangered Wildlife in Canada. Ottawa. xiv + 84 pp. <https://wildlife-species.canada.ca/species-risk-registry/virtual_sara/files/cosewic/sr_ours_grizz_bear_1012_e.pdf>

COSEWIC. 2012h. COSEWIC assessment and status report on the Bull Trout *Salvelinus confluentus* in Canada. Committee on the Status of Endangered Wildlife in Canada. Ottawa. iv + 103 pp. <https://wildlife-species.canada.ca/species-risk-registry/virtual_sara/files/cosewic/sr_omble_tete_plat_bull_trout_1113_e.pdf>

COSEWIC. 2012i. COSEWIC assessment and status report on the American Eel *Anguilla rostrata* in Canada. Committee on the Status of Endangered Wildlife in Canada. Ottawa. x ii + 109 pp. <https://wildlife-species.canada.ca/species-risk-registry/virtual_sara/files/cosewic/sr_anguille_amer_eel_1012_e.pdf>

COSEWIC. 2011a. COSEWIC assessment and status report on the Macropis Cuckoo Bee *Epeoloides pilosulus* in Canada. [Committee on the Status of Endangered Wildlife in Canada](http://www.sararegistry.gc.ca/default_e.cfm). Ottawa. ix + 25 pp. <https://wildlife-species.canada.ca/species-risk-registry/virtual_sara/files/cosewic/sr_macropis_cuckoo_bee_0911_eng.pdf>

COSEWIC. 2011b. COSEWIC assessment and status report on the Barn Swallow *Hirundo rustica* in Canada. Committee on the Status of Endangered Wildlife in Canada. Ottawa. ix + 37 pp. <https://wildlife-species.canada.ca/species-risk-registry/virtual_sara/files/cosewic/sr_barn_swallow_0911_eng.pdf>

COSEWIC. 2011c. COSEWIC assessment and status report on the Eastern Meadowlark *Sturnella magna* in Canada. Committee on the Status of Endangered Wildlife in Canada. Ottawa. x + 40 pp. <https://wildlife-species.canada.ca/species-risk-registry/virtual_sara/files/cosewic/sr_eastern_meadowlark_0911_eng.pdf>

COSEWIC. 2011d. COSEWIC assessment and status report on the Collared Pika *Ochotona collaris* in Canada. Committee on the Status of Endangered Wildlife in Canada. Ottawa. x + 50 pp. <https://wildlife-species.canada.ca/species-risk-registry/virtual_sara/files/cosewic/sr_collared_pika_collier_0912_e.pdf>

COSEWIC. 2011e. COSEWIC assessment and status report on the Silver Lamprey, Great Lakes - Upper St. Lawrence populations and Saskatchewan - Nelson Rivers populations *Ichthyomyzon unicuspis* in Canada. Committee on the Status of Endangered Wildlife in Canada. Ottawa. xiii + 55 pp. <https://wildlife-species.canada.ca/species-risk-registry/virtual_sara/files/cosewic/sr_silver_lamprey_0911_eng.pdf>

COSEWIC. 2011f. Designatable Units for Caribou (*Rangifer tarandus*) in Canada. Committee on the Status of Endangered Wildlife in Canada. Ottawa. 88pp. <https://www.canada.ca/content/dam/eccc/migration/cosewic-cosepac/4e5136bf-f3ef-4b7a-9a79-6d70ba15440f/cosewic_caribou_du_report_23dec2011.pdf>

COSEWIC. 2010a. COSEWIC assessment and status report on the Sprague’s Pipit *Anthus spragueii* in Canada. Committee on the Status of Endangered Wildlife in Canada. Ottawa. ix + 34 pp. <https://www.canada.ca/en/environment-climate-change/services/species-risk-public-registry/cosewic-assessments-status-reports/sprague-pipit-2010.html#_docInfo>

COSEWIC. 2010b. COSEWIC assessment and status report on the Bobolink *Dolichonyx oryzivorus* in Canada. Committee on the Status of Endangered Wildlife in Canada. Ottawa. vi + 42 pp. <https://wildlife-species.canada.ca/species-risk-registry/virtual_sara/files/cosewic/sr_Bobolink_0810_e.pdf>

COSEWIC. 2010c. COSEWIC assessment and status report on the Whooping Crane *Grus americana* in Canada. Committee on the Status of Endangered Wildlife in Canada. Ottawa. x + 36 pp. <https://wildlife-species.canada.ca/species-risk-registry/virtual_sara/files/cosewic/sr_Whooping%20Crane_0810_e.pdf>

COSEWIC. 2010d. COSEWIC assessment and status report on the Pitcher’s Thistle *Cirsium pitcheri* in Canada. Committee on the Status of Endangered Wildlife in Canada. Ottawa. x + 32 pp. <https://wildlife-species.canada.ca/species-risk-registry/virtual_sara/files/cosewic/sr_pitchers_thistle_0911_eng.pdf>

COSEWIC. 2010e. COSEWIC assessment and status report on the Dolly Varden *Salvelinus malma malma* (Western Arctic populations) in Canada. Committee on the Status of Endangered Wildlife in Canada. Ottawa. x + 65 pp. <https://wildlife-species.canada.ca/species-risk-registry/virtual_sara/files/cosewic/sr_dolly_varden_0911_eng.pdf>

COSEWIC. 2010f. COSEWIC assessment and status report on the Atlantic Salmon *Salmo salar* (Nunavik population, Labrador population, Northeast Newfoundland population, South Newfoundland population, Southwest Newfoundland population, Northwest Newfoundland population, Quebec Eastern North Shore population, Quebec Western North Shore population, Anticosti Island population, Inner St. Lawrence population, Lake Ontario population, Gaspé-Southern Gulf of St. Lawrence population, Eastern Cape Breton population, Nova Scotia Southern Upland population, Inner Bay of Fundy population, Outer Bay of Fundy population) in Canada. Committee on the Status of Endangered Wildlife in Canada. Ottawa. xlvii + 136 pp.. <https://wildlife-species.canada.ca/species-risk-registry/virtual_sara/files/cosewic/sr_Atlantic_Salmon_2011a_e.pdf>

COSEWIC. 2009a COSEWIC assessment and update status report on the Northern Leopard Frog *Lithobates pipiens*, Rocky Mountain population, Western Boreal/Prairie populations and Eastern populations, in Canada. Committee on the Status of Endangered Wildlife in Canada. Ottawa. vii + 69 pp. <https://wildlife-species.canada.ca/species-risk-registry/virtual_sara/files/cosewic/sr_northern_leopard_frog_0809i_e.pdf>

COSEWIC. 2009b. COSEWIC assessment and status report on the Whip-poor-will *Caprimulgus vociferous* in Canada. Committee on the Status of Endangered Wildlife in Canada. Ottawa. vi + 28 pp. (www.sararegistry.gc.ca/status/status_e.cfm). <https://www.canada.ca/en/environment-climate-change/services/species-risk-public-registry/cosewic-assessments-status-reports/whip-poor-will-2009.html>

COSEWIC. 2009c. COSEWIC assessment and status report on the Bicknell's Thrush *Catharus bicknelli* in Canada. Committee on the Status of Endangered Wildlife in Canada. Ottawa. vii + 44 pp. <https://wildlife-species.canada.ca/species-risk-registry/virtual_sara/files/cosewic/sr_Bicknell's%20Thrush_0810_e.pdf>

COSEWIC. 2009d. COSEWIC assessment and status report on the Yellow Rail Coturnicops noveboracensis in Canada. Committee on the Status of Endangered Wildlife in Canada. Ottawa. vii + 32 pp. <https://wildlife-species.canada.ca/species-risk-registry/virtual_sara/files/cosewic/sr_Yellow%20Rail_0810_e.pdf>

COSEWIC. 2009e. COSEWIC assessment and update status report on the Least Bittern *Ixobrychus exilis* in Canada. Committee on the Status of Endangered Wildlife in Canada. Ottawa. vi + 36 pp. <https://wildlife-species.canada.ca/species-risk-registry/virtual_sara/files/cosewic/sr_least_bittern_0809_e.pdf>

COSEWIC. 2009f. COSEWIC assessment and status report on the Eskimo Curlew *Numenius borealis* in Canada. Committee on the Status of Endangered Wildlife in Canada. Ottawa. vii + 32 pp. <https://wildlife-species.canada.ca/species-risk-registry/virtual_sara/files/cosewic/sr_Eskimo%20Curlew_0810_e.pdf>

COSEWIC. 2009g. COSEWIC assessment and status report on the Horned Grebe *Podiceps auritus*, Western population and Magdalen Islands population, in Canada. Committee on the Status of Endangered Wildlife in Canada. Ottawa. vii + 42 pp. <https://wildlife-species.canada.ca/species-risk-registry/virtual_sara/files/cosewic/sr_horned_grebe_0809_e.pdf>

COSEWIC. 2009h. COSEWIC assessment and update status report on the Bigmouth Buffalo *Ictiobus cyprinellus*, Great Lakes - Upper St. Lawrence populations and Saskatchewan - Nelson River populations, in Canada. Committee on the Status of Endangered Wildlife in Canada. Ottawa. vii + 40 pp. <https://wildlife-species.canada.ca/species-risk-registry/virtual_sara/files/cosewic/sr_bigmouth_buffalo_0809_e.pdf>

COSEWIC.2008a.COSEWIC assessment and update status report on the Short-eared Owl *Asio flammeus* in Canada. Committee on the Status of Endangered Wildlife in Canada. Ottawa. vi + 24 pp. <https://wildlife-species.canada.ca/species-risk-registry/virtual_sara/files/cosewic/sr_shorteared_owl_0808_e.pdf>

COSEWIC. 2008b. COSEWIC assessment and status report on the Canada Warbler *Wilsonia canadensis* in Canada. Committee on the Status of Endangered Wildlife in Canada. Ottawa. vi + 35 pp. <https://wildlife-species.canada.ca/species-risk-registry/virtual_sara/files/cosewic/sr_canada_warbler_0808_e.pdf>

COSEWIC. 2008c. COSEWIC assessment and status report on the Snapping Turtle *Chelydra serpentina* in Canada. Committee on the Status of Endangered Wildlife in Canada. Ottawa. vii + 47 pp. <https://wildlife-species.canada.ca/species-risk-registry/virtual_sara/files/cosewic/sr_snapping_turtle_0809_e.pdf>

COSEWIC 2007a. COSEWIC assessment and status report on the Red Knot *Calidris canutus* in Canada. Committee on the Status of Endangered Wildlife in Canada. Ottawa. vii + 58 pp. <https://wildlife-species.canada.ca/species-risk-registry/virtual_sara/files/cosewic/sr_calidris_canutus_e.pdf>

COSEWIC 2007b. COSEWIC assessment and update status report on the northern brook lamprey *Ichthyomyzon fossor* (Great Lakes – Upper St. Lawrence populations and Saskatchewan – Nelson population) in Canada. Committee on the Status of Endangered Wildlife in Canada. Ottawa. vi + 30 pp. <https://wildlife-species.canada.ca/species-risk-registry/virtual_sara/files/cosewic/sr_ichthyomyzon_fossor_e.pdf>

COSEWIC 2004. COSEWIC assessment and update status report on the Loggerhead Shrike *excubitoride*s subspecies *Lanius ludovicianus* in Canada. Committee on the Status of Endangered Wildlife in Canada. Ottawa. vi + 24 pp. <https://wildlife-species.canada.ca/species-risk-registry/virtual_sara/files/cosewic/sr_loggerhead_shrike_e.pdf>

COSEWIC 2003. COSEWIC assessment and update status report on the Shortjaw cisco *Coregonus zenithicus*. Committee on the Status of Endangered Wildlife in Canada. Ottawa. viii + 19 pp. <https://wildlife-species.canada.ca/species-risk-registry/virtual_sara/files/cosewic/sr_shortjaw_cisco_e.pdf>

COSEWIC. 2000a. COSEWIC assessment and status report on the Barrow’s Goldeneye *Bucephala islandica*, Eastern population, in Canada. Committee on the Status of Endangered Wildlife in Canada. Ottawa. vii + 65 pp. <https://wildlife-species.canada.ca/species-risk-registry/virtual_sara/files/cosewic/Barrow%E2%80%99s%20Goldeneye_2000_e.pdf>

COSEWIC 2000b. COSEWIC assessment and update status report on Greater Prairie-Chicken *Tympanuchus cupido* in Canada. Committee on the Status of Endangered Wildlife in Canada. Ottawa. vi + 7 pp. <https://wildlife-species.canada.ca/species-risk-registry/virtual_sara/files/cosewic/sr_greater_prairie_chicken_0500_e.pdf>

COSEWIC 2000c. COSEWIC assessment and update status report on the American ginseng *Panax quinquefolius* in Canada. Committee on the Status of Endangered Wildlife in Canada. Ottawa. vii + 17 pp. <https://wildlife-species.canada.ca/species-risk-registry/virtual_sara/files/cosewic/sr1_americanginseng_nl_e.pdf>

Department of Fisheries and Oceans. 2017. Fisheries and Oceans Canada Species at Risk Distribution (Range) [Data set]. Government of Canada. Accessed 2019-06-21. <https://open.canada.ca/data/en/dataset/e0fabad5-9379-4077-87b9-5705f28c490b>

Department of Fisheries and Oceans and Québec Ministère des Ressources naturelles et de la Faune (DFO and MNRF). 2008. Conservation Status Report, Atlantic Salmon in Atlantic Canada and Quebec: PART I – Species Information. Can. MS Rep. Fish. Aquat. Sci. No. 2861, 208 p. <https://waves-vagues.dfo-mpo.gc.ca/Library/335625.pdf>

Environment Canada. 2011. Management Plan for Fernald’s Milk-vetch (*Astragalus robbinsiivar. fernaldii*) in Canada. Species at Risk Act Management Plan Series, Environment Canada, Ottawa, iv + 14 pp. <https://wildlife-species.canada.ca/species-risk-registry/virtual_sara/files/plans/mp_fernald's_milk_vetch_0811_eng.pdf>

Environment and Climate Change Canada. 2016. Range map extents, species at risk, Canada. Government of Canada. Open Government Dataset. <https://open.canada.ca/data/en/dataset/d00f8e8c-40c4-435a-b790-980339ce3121>

Supplemental Table S2. Protected areas management categories for the International Union for Conservation of Nature used by Canada to report on Aichi 11 goals (taken from the 2019 Canadian Protected and Conserved Areas Database (CPCAD)).

| IUCN category | Area (km^2^) | Example of type |
| --- | --- | --- |
| Ia | 3,182 | Mccusker River (Ecological Reserve) |
| Ib | 79,478 | Edéhzhíe Protected Area (National Wildlife Area) |
| II | 133,056 | Proposed Albanel-Témiscamie-Otish Biodiversity Reserve  (Proposed Biodiversity Reserve) |
| III | 405 | Little Limestone Lake Provincial Park (Provincial Park) |
| IV | 6,954 | Weeskayjahk Ohtahzhoganeeng (Provincial Park) |
| V | 194 | Candle Lake Provincial Park (Recreation Park) |
| VI | 5,775 | Saoyú-Ɂehdacho National Historic Site Of Canada (National Historic Site) |
| Other^1^ | 16,368 | Cat Lake-Slate Falls Dedicated Protected Area (Protected Area - Far North) |
|  |  | Johnny Hoe River (Conservation zone in regional land use plan) |

^1^Other Effective Area-Based Conservation Measure ([OECMs](https://www.iucn.org/commissions/world-commission-protected-areas/our-work/oecms#:~:text=%E2%80%98Other%20effective%20area-based%20conservation%20measures%E2%80%99%20%28OECMs%29%20is%20a,through%20a%20WCPA%20Task%20Force%20on%20OECMs%20%28Reports%29.)), defined as areas other than protected areas that achieve positive and sustained long-term outcomes for conservation of biodiversity.

Supplemental Fig. S1. Seven unique species occupying the distribution of boreal caribou. These species were selected because they occupied a small fraction of the boreal caribou distribution (x-axes: < 20%) despite most of their Canadian inland distribution occurring within the distribution of boreal caribou (y-axis; ≥ 50%).


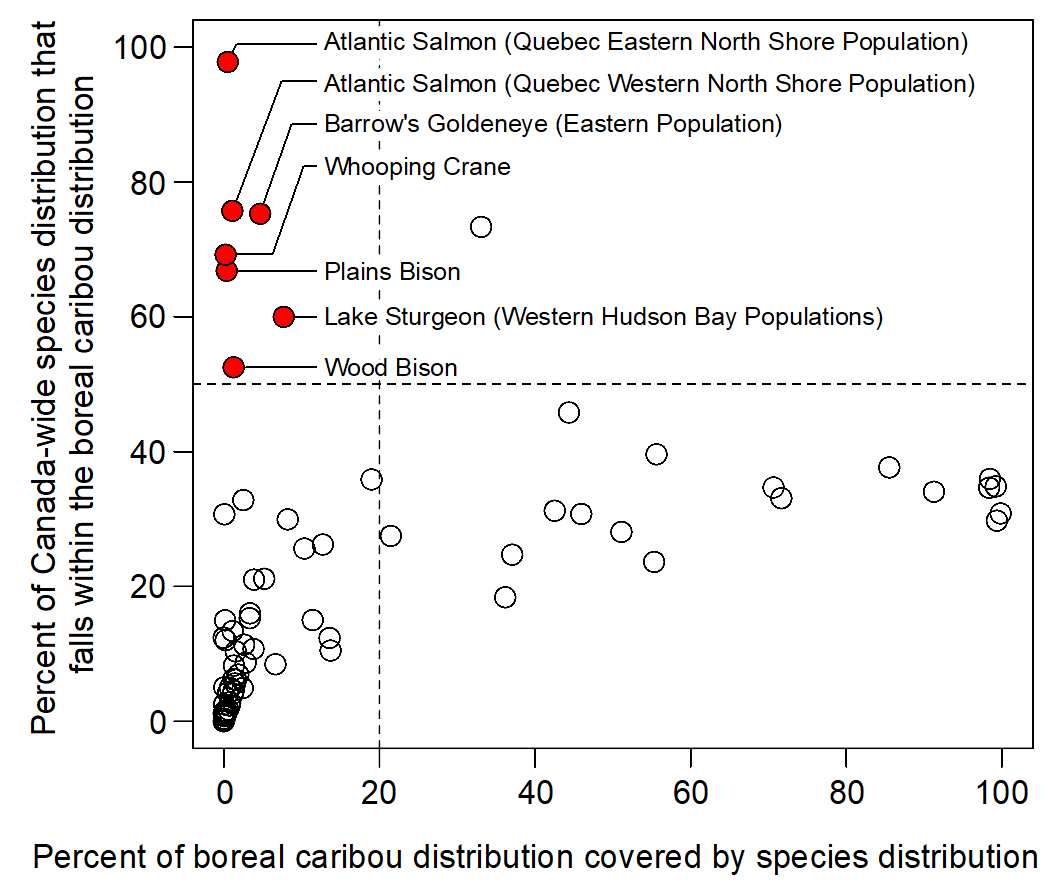

Supplement: Supplementary file 1 — Supplementary Information. [file 41598_2022_21476_MOESM1_ESM.docx]
